# Supplementary material for: Evidence for the stabilization of FeN4 sites by Pt particles during acidic oxygen reduction
Source: Nat Commun. 2025 Jul 11;16:6404. doi: 10.1038/s41467-025-61806-x (PMC12246215; doi:10.1038/s41467-025-61806-x)
Supplement: Supplementary file 1 — Supplementary Information [file 41467_2025_61806_MOESM1_ESM.pdf]

Supplementary information for

Evidence for the stabilization of FeN_4 sites by Pt particles during acidic oxygen reduction

Nicolas A. Ishiki[1,2,3‡], Keyla Teixeira Santos[1,4‡], Nicolas Bibent[5], Kavita Kumar[1], Ina Reichmann[6,7], Yu-Ping Ku[6,7], Tristan Asset[5,8], Laetitia Dubau[1], Michel Mermoux[1], Hongxin Ge[9], Sandrine Berthon-Fabry[9], Viktoriia A. Saveleva[10,11], Vinod K. Paidi[10], Pieter Glatzel[10], Andrea Zitolo[12], Tzonka Mineva[5], Hazar Guesmi[5], Serhiy Cherevko[6], Edson A. Ticianelli[2], Frédéric Maillard[1*], and Frédéric Jaouen[5*]

[1]Univ. Grenoble Alpes, Univ. Savoie Mont Blanc, CNRS, Grenoble INP, LEPMI, 38000 Grenoble, France.

[2]Instituto de Química de São Carlos (IQSC), Universidade de São Paulo, Av. Trabalhador São-Carlense, 400, CP 780, São Carlos, SP, Brazil.

[3]Present address: Université Paris Cité, CNRS, ITODYS, 75013, Paris, France.

[4]Present address: Instituto de Química, Universidade Estadual de Campinas (UNICAMP), R. Monteiro Lobato, 270, 13083-970, Campinas, SP, Brazil.

[5]ICGM, Univ. Montpellier, CNRS, ENSCM, 1919 route de Mende, 34293 Montpellier, France.

[6]Forschungszentrum Jülich GmbH, Helmholtz-Institute Erlangen-Nürnberg for Renewable Energy (IET-2), Cauerstraße 1, 91058 Erlangen, Germany.

[7]Friedrich-Alexander-Universität Erlangen-Nürnberg, Department of Chemical and Biological Engineering, Cauerstraße 1, 91058 Erlangen, Germany.

[8]Institut de Chimie et Procédés pour l'Energie, l'Environnement et la Santé, UMR 7515 CNRS-University of Strasbourg, 25 rue Becquerel 67087, Strasbourg Cedex, France.

[9]MINES Paris, PSL University PERSEE - Centre procédés, énergies renouvelables et systèmes énergétiques, CS 10207 rue Claude Daunesse, 06904 Sophia Antipolis Cedex, France.

[10]ESRF, The European Synchrotron, 71 Avenue des Martyrs, 38000, Grenoble, France.

[11]Present address: Li², joint laboratory of Blue Solutions, LEPMI and Grenoble INP, 1025 rue de la Piscine, 38610 Gières, France.

[12]Synchrotron SOLEIL, L'Orme des Merisiers, BP 48 Saint Aubin, 91192 Gif-sur-Yvette, France.

‡These authors contributed equally to this work.

* Corresponding authors.

E-mail address: frederic.jaouen@umontpellier.fr, frederic.maillard@grenoble-inp.fr.

Table of Contents

Supplementary Information Notes 2

Supplementary Information Scheme 6

Supplementary Information Figures 7

Supplementary Information Tables 34

Supplementary Information References 45

Supplementary Information Notes

Supplementary Note 1 | Estimation of the Pt specific contribution to ORR activity in Pt/N–C_{Aero} and Pt/Fe–N–C_{Aero}

Starting from the mass activity (MA) at 0.8 V (Pt/N–C_{Aero} has a MA value of 1.19 A g^{-1}_{powder}), the MA numbers at 0.8 V *vs*. RHE can simply be multiplied by 100 to convert them from A g^{-1} powder to A g^{-1}_{Pt}, since the Pt loading is *ca*. 1 wt. % Pt. This leads to MA numbers of 119 A g^{-1}_{Pt} for Pt/N–C_{Aero}, at 0.8 V *vs*. RHE. However, as the MA of ORR-active Pt is usually reported at 0.9 V *vs*. RHE, it is more meaningful to convert this number to the expected MA at 0.9 V, and this was done assuming a Tafel slope of 75 mV/decade (which is also the experimental Tafel slope observed). According to Tafel law and classical math, this leads to a conversion factor of / 21.45 to convert MA values from 0.8 V to 0.9 V, *i.e.* 5.5 A g^{-1}_{Pt} for Pt/N–C_{Aero} at 0.9 V *vs*. RHE. For Pt in Pt/Fe–N–C_{Aero}, assuming the difference in MA values between Pt/Fe–N–C_{Aero} and Fe–N–C_{Aero} is representative of the Pt contribution towards ORR activity, (3.95-2.71 = 1.24 A g^{-1}_{powder}, at 0.8 V), one can then calculate a possible MA contribution of Pt present in Pt/Fe–N–C_{Aero} to be *ca*. 124 A g^{-1}_{Pt} at 0.8 V *vs*. RHE, corresponding to 124/21.45 = 5.8 A g_{Pt}^{-1} at 0.9 V *vs*. RHE.

Supplementary Note 2 | Calculation of the diffusion-limited current density during HPRR measurement

The theoretical diffusion-limited current density expected during RDE measurement looking at the two-electron electro-reduction activity of H_2O_2 to H_2O is calculated according to Levich equation

$$j_L = (0.620) \times n \times F \times D^{\frac{2}{3}} \times \omega^{\frac{1}{2}} \times \nu^{\left(-\frac{1}{6}\right)} \times C$$

Where j_L is in A·cm^{-2}, n is the number of electrons exchanged for each reactant molecule (n = 2 for H_2O_2 to H_2O), F is Faraday's constant (96485.3 C·mol^{-1}), D is the H_2O_2 diffusion coefficient in the electrolyte (1.3·10^{-5} cm^2·s^{-1}),[1] ω is the angular rotation rate (167.55 rad·s^{-1}, corresponding to 1600 rpm), ν is the kinematic viscosity of the 0.1 M H_2SO_4 electrolyte (10^{-2} cm^2·s^{-1})[1] and C is the H_2O_2 bulk concentration in the electrolyte (10^{-5} mol·cm^{-3}, corresponding to 10 mM). Substituting these values in the above equation gives j_L = 0.01845 A cm^{-2}, or 18.45 mA cm^{-2}. This is the current density at the diffusional limit that should be obtained, if the electrode were being limited solely by the diffusion of H_2O_2.

Supplementary Note 3 | Scanning Gas-diffusion electrode half-cell coupled to inductively plasma mass spectrometry (S-GDE-ICP-MS) methods

Before the preparation of the standard solutions for S-GDE-ICP-MS calibration, two intermediate ones were prepared, each containing 1 $mg_{Fe}\cdot L^{-1}$ and 1 $mg_{Pt}\cdot L^{-1}$. The solutions were obtained by diluting the Merck Centripur ICP standards (1000 $mg_{Fe}\cdot L^{-1}$ in 2–3 % HNO_3, and 1000 $mg_{Pt}\cdot L^{-1}$ in 7 % HCl, respectively) with 1 wt. % HNO_3. Next, with these two intermediate standard solutions and the electrolyte, 0.1 M $HClO_4$ (ROTIPURAN®Supra, ROTH), the three standard solutions were prepared. For example, the first standard solution comprised 1 $\mu g\cdot L^{-1}$ of Fe and 1 $\mu g\cdot L^{-1}$ of Pt. It was prepared by mixing 10 µL of the intermediate standard solution with 1 $mg_{Fe}\cdot L^{-1}$, 10 µL of the other solution with 1 $mg_{Pt}\cdot L^{-1}$, and 9.98 mL of the electrolyte.

Supplementary Note 4 | Performance at high current density of $Fe_{0.5}$ *vs.* Pt/$Fe_{0.5}$ beginning-of-test (BoT) curves

The proton-exchange-membrane fuel cell polarization curve shown in Fig. 6c for $Fe_{0.5}$ is from our previous work (Ref. [2]) and was obtained with slightly different gas-diffusion layer (GDL) and cathode preparation. However, the polarization curve for a recent $Fe_{0.5}$ batch with its GDL and cathode prepared identically as the Pt/$Fe_{0.5}$ cathode shown in Fig. 6c also underperformed the Pt/$Fe_{0.5}$ at high current density (Supplementary Fig. 20).

Supplementary Note 5 | Discussion of PEMFC results shown in Supplementary Fig. 21

Additional PEMFC measurements were carried out with a commercial Pt/C catalyst (40 wt. % Pt on Vulcan XC72) and preparing ultrathin cathode layers to result in a Pt loading of only 40 $\mu g_{Pt}\cdot cm^{-2}_{geo}$ at the cathode, as a comparison to the performance obtained with cathodes based on Pt/Fe–N–C_{Aero} and Pt/N–C_{Aero}, with a total cathode catalyst loading of 4 $mg_{powder}\cdot cm^{-2}_{geo}$, that also comprise *ca.* 40 $\mu g_{Pt}\cdot cm^{-2}_{geo}$ (1 wt. % Pt on Fe–N–C_{Aero} and N–C_{Aero} in the Pt/Fe–N–C_{Aero} and Pt/N–C_{Aero} materials). The BoT performance for Pt/C is very low, with an activity at 0.8 V as low as that of Pt/N–C_{Aero}. However, after the potential hold at 0.5 V, the performance increases dramatically and the Pt/C cathode reaches an activity at 0.8 V comparable to those of Fe–N–C_{Aero} and Pt/Fe–N–C_{Aero} at BoT. It is well known that state-of-art Pt/C cathodes require a break-in process in PEMFC during which the cell performance increases. In the present case, the current density of the Pt/C cathode increased during the first 10 h of the voltage hold at 0.5 V, then stabilized. Such a behavior is the fingerprint of active platinum cathodes. In contrast,

the activity at 0.8 V and cell performance of the Pt/N–C_{Aero} cathode only increased marginally during the same potential hold, highlighting the low ORR activity of Pt NPs deposited at the low content of 1 wt. % on the N–C_{Aero} support. We stress that the same activity at 0.8 V of the Pt/C cathode at EoT and of the Pt/Fe–N–C_{Aero} at BoT cannot be interpreted as a Pt-driven activity for the Pt/Fe–N–C_{Aero}: First, the same activity at 0.8 V is also observed for the Pt-free Fe–N–C_{Aero} cathode at BoT, inferring that the activity of Pt/Fe–N–C_{Aero} should have been significantly higher, if the Pt in Pt/Fe–N–C_{Aero} was highly active. Second, the Pt/Fe–N–C_{Aero} does not behave as the Pt/C material in the sense that it overall loses a small amount of activity and performance after the potential hold (see Figure 5a). The limited improvement in current density from *ca.* 900 to *ca.* 950 mA cm^{-2} observed during the first 2 h of the potential hold of Pt/Fe–N–C_{Aero} (see Figure 5b) may however be interpreted as the activity break-in of the Pt NPs in Pt/Fe–N–C_{Aero}. If this is true, it shows that the contribution of Pt to the overall activity and performance of Pt/Fe–N–C_{Aero} is not zero, but is minor, in line with all the other electrochemical results of the present study. Last, the lower performance at high current density of the ultrathin Pt/C cathode compared to thick Fe–N–C_{Aero} and Pt/Fe–N–C_{Aero} layers may be assigned to water flooding. While the thick Fe–N–C_{Aero} and Pt/Fe–N–C_{Aero} layers are not optimum for O_2 and proton transport, the thin Pt/C layer results in a low absolute volume of pores, and the high amount of water produced by the ORR at high current density can easily flood these pores. The carbon loading (driving the cathode active layer thickness) is only 60 $\mu g_C \cdot cm^{-2}_{geo}$ for the Pt/C cathode, but *ca.* 4 $mg_C \cdot cm^{-2}_{geo}$ (neglecting the relatively low Fe and N contents in Fe–N–C) in the Fe–N–C_{Aero} and Pt/Fe–N–C_{Aero} layers. Thus, one can estimate that the Pt/C layer is *ca.* 66 times thinner than the Fe–N–C_{Aero} and Pt/Fe–N–C_{Aero} layers.

Supplementary Note 6 | Calculating the number of Pt particles on Pt/Fe–N–C catalysts

The input variables are the mass of Pt deposited on Fe–N–C (m_{Pt}) and the diameter of the Pt particles (*d*, supposed to be spherical and monodisperse size). The calculations are performed starting from a mass of Fe–N–C of 1 g and, assuming a given site density of FeN_4 sites per gram of Fe–N–C, the number of sites will be the same on the final Pt/Fe–N–C hybrid (with total mass 1 + m_{Pt}). A range of m_{Pt} values (in gram) is then considered to be deposited on 1-gram Fe–N–C. From this, the weight percentage of Pt on the final Pt/Fe–N–C hybrid is calculated from:

$Pt\ wt.\ \% = 100\ m_{Pt} / (1 + m_{Pt})$

The range of calculated of Pt wt. % forms the x-axis in Supplementary Fig. 23. Then, the mass of a single Pt particle is calculated from the Pt particle diameter d and the Pt density (ρ):

$$m_{\text{Pt-Particle}} = \rho \cdot V = \rho\ \pi\ d^3 / 6$$

with ρ = 21.45 g cm^{-3}, and d value input in cm.

The number of Pt particles corresponding to a given value of m_{Pt} deposited on Fe–N–C is then obtained from:

$$N_{\text{Pt-Particle}} = m_{\text{Pt}} / m_{\text{Pt-Particle}}$$

And the ratio of FeN_4 sites number present in Pt/Fe–N–C to the number of Pt particles deposited on Fe–N–C is given by

$$R = \text{SD} / N_{\text{Pt-Particle}}$$

Where SD is the hypothesized site density of FeN_4 sites per gram Fe–N–C.

While the assumption of spherical Pt particle geometry is not strictly correct, this does not induce strong deviation with more realistic and complex geometries of real Pt particles. For example, with our calculations a Pt sphere of 1.7 nm diameter results in a particle mass that in turn corresponds to 170 Pt atoms. This is comparable to 201 Pt atoms calculated for a Pt particle with realistic truncated octahedral shape and average particle diameter of 1.75 nm.[3]

The baseline SD value of $3 \cdot 10^{19}$ sites / g corresponds to the SD value experimentally measured on $Fe_{0.5}$ catalyst.[4] The higher value of SD of 10^{20} sites g^{-1} is close to the highest experimentally reported SD values for Fe–N–C materials.[5,6]

Supplementary Information Scheme

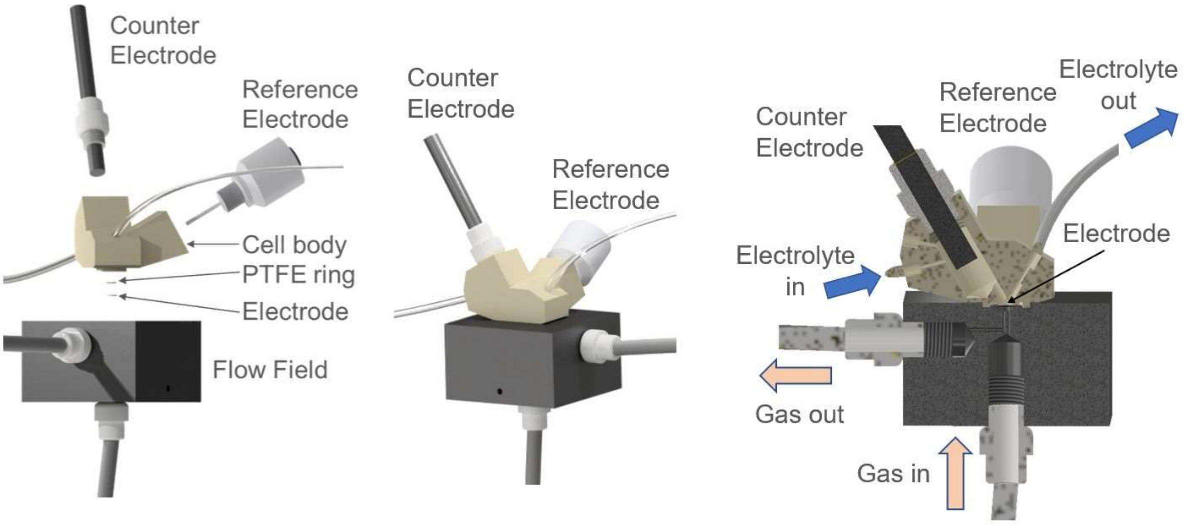

Supplementary Scheme 1 | Scanning GDE half-cell with online ICP-MS

Design of the S-GDE. From left to right: disassembled parts of the S-GDE, assembled parts of the S-GDE, and cross section view of the cell and flow field to visualize the electrolyte flow and gas flow. Adapted from Fig. 1 in [7].

Supplementary Information Figures

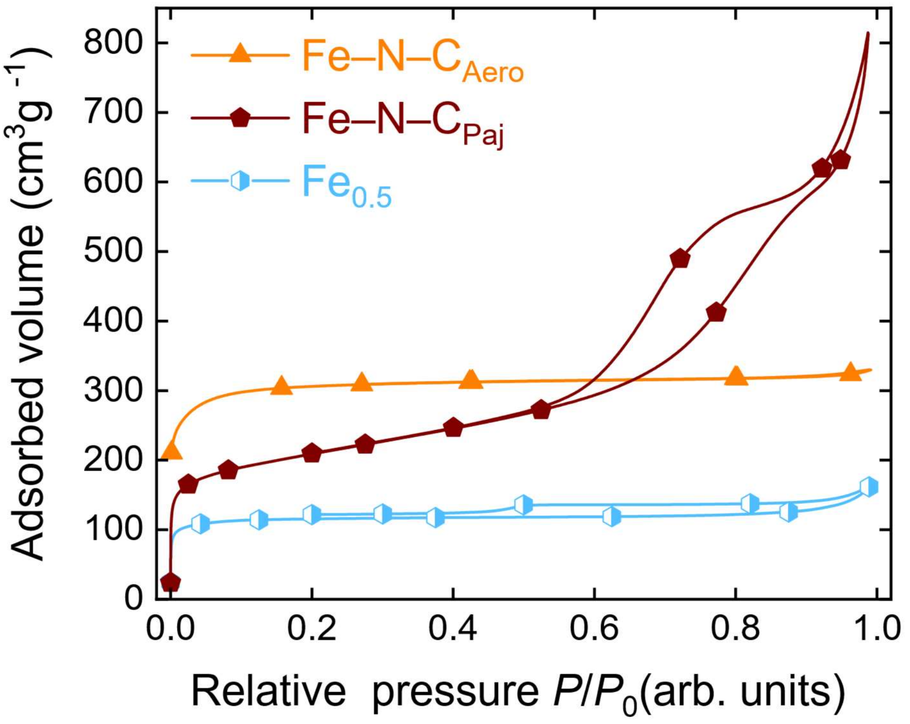

Supplementary Fig. 1 | Nitrogen sorption isotherms of pristine Fe–N–C powders.

The BET specific surface area as well as pore volumes derived from the analysis of the adsorption branch of each Fe–N–C catalyst are reported in Supplementary Table 1. Source data are provided as a Source Data file.

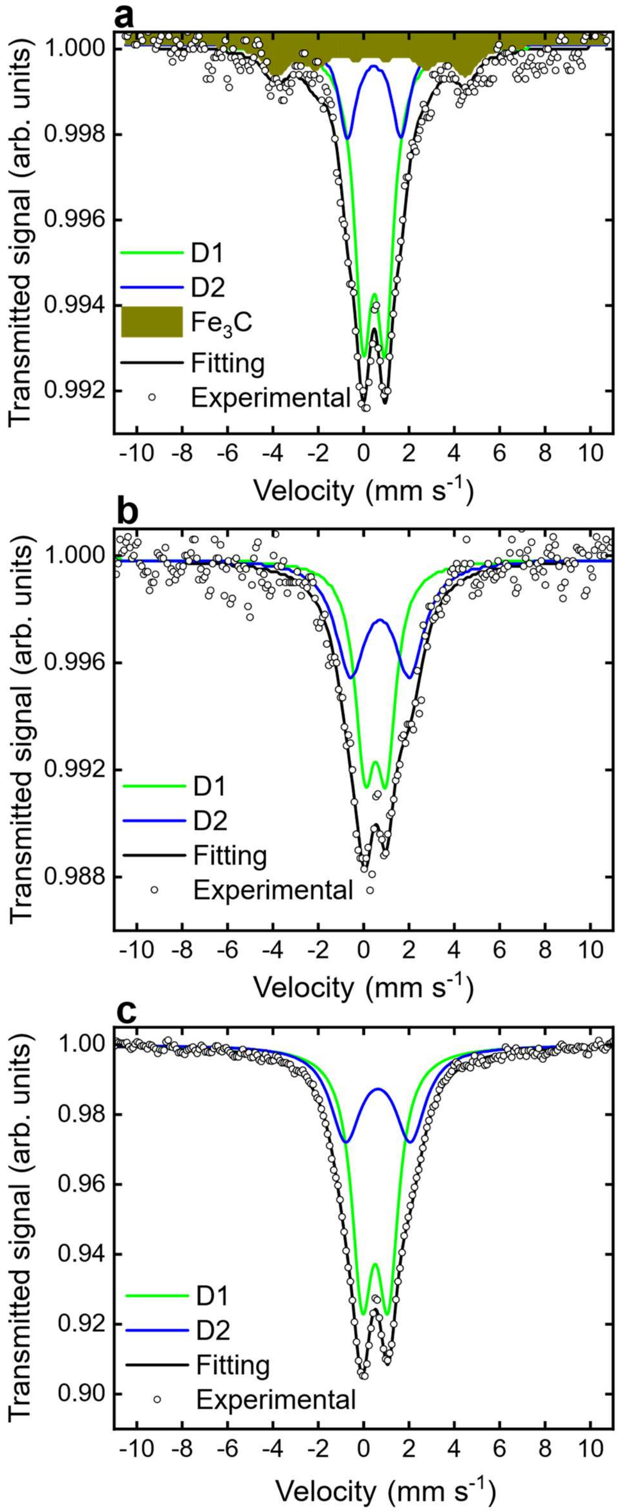

Supplementary Fig. 2 | ^{57}Fe Mössbauer spectra of pristine Fe–N–C powders.

(a) Spectrum of Fe–N–C_{Aero}, **(b)** spectrum of Fe–N–C_{Paj}, **(c)** spectrum of $Fe_{0.5}$. The spectral parameters obtained from the fittings are presented in Supplementary Table 2. The spectra were measured in natural air at -268 °C. Source data are provided as a Source Data file.

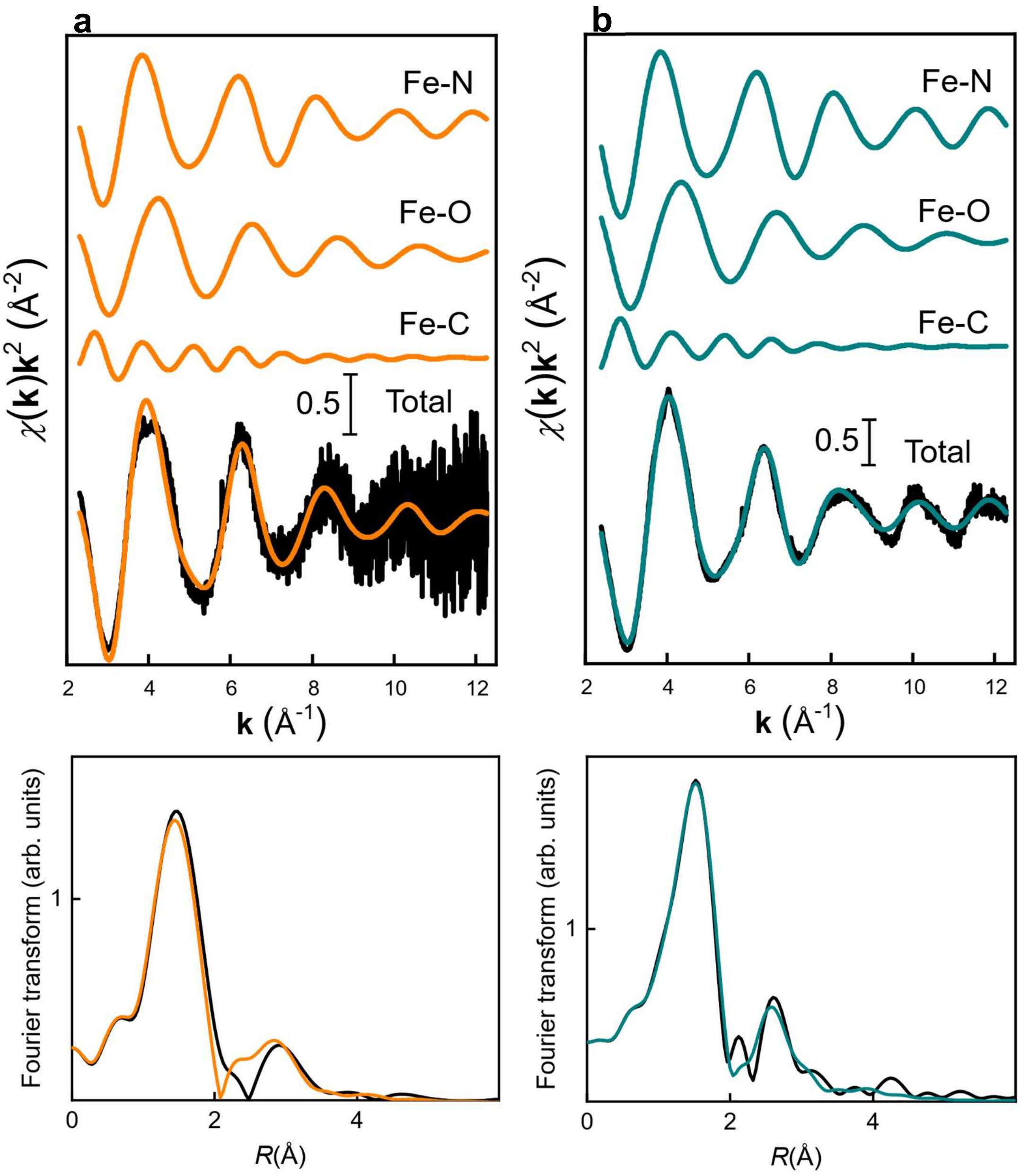

Supplementary Fig. 3 | Fe *K*-edge EXAFS analysis of pristine Fe–N–C powders.

(a) Fe–N–C$_{Aero}$ and **(b)** Pt/Fe–N–C$_{Aero}$. Upper panel: Fe-N, Fe-O and Fe-C $\gamma^{(2)}$ two-body signals included in the fit, the total signal (orange or dark cyan line) superimposed to the experimental one (black line). Lower panel: the fit in the Fourier transformed space. Source data are provided as a Source Data file.

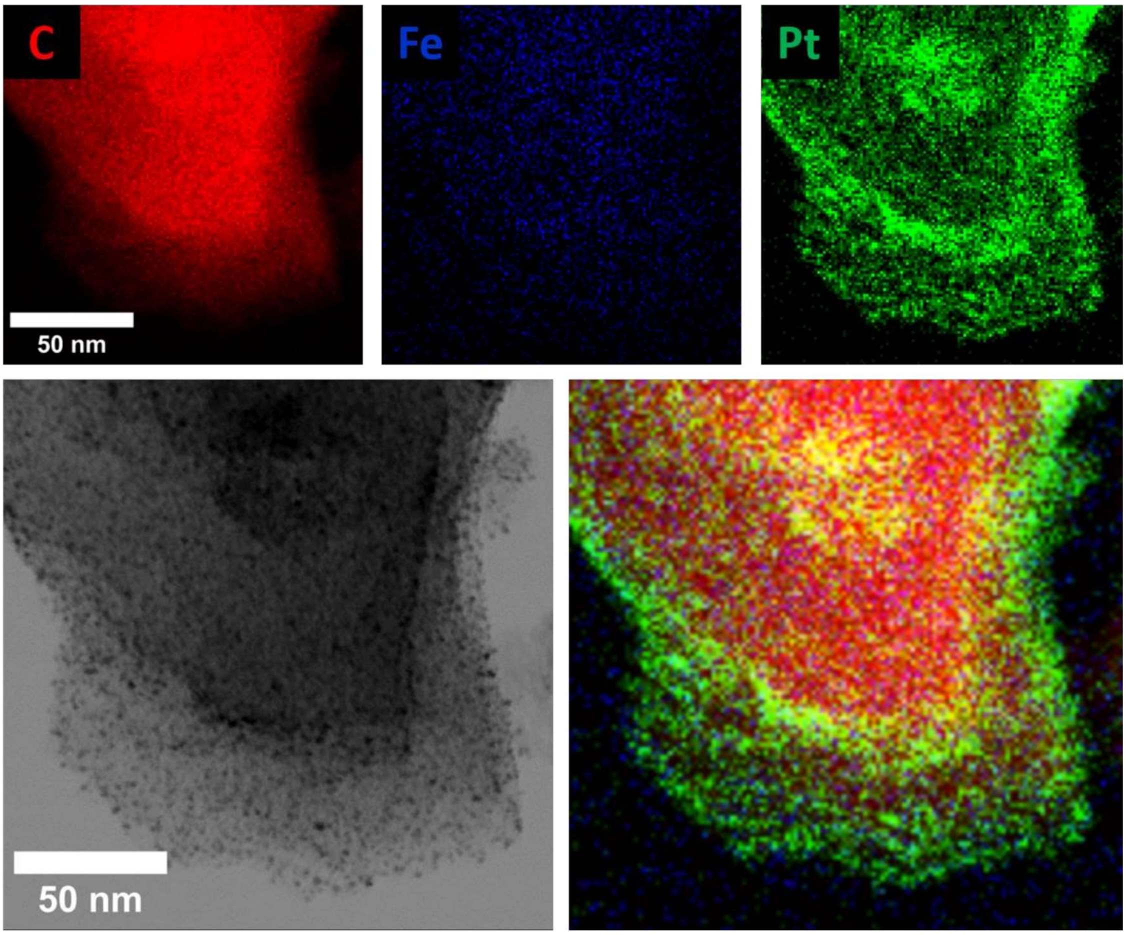

Supplementary Fig. 4 | Pt and Fe dispersion in Pt/Fe–N–C_{Aero}

Scanning transmission electron microscopy measurements of Pt/Fe–N–C_{Aero} coupled with energy dispersive X-ray spectroscopy.

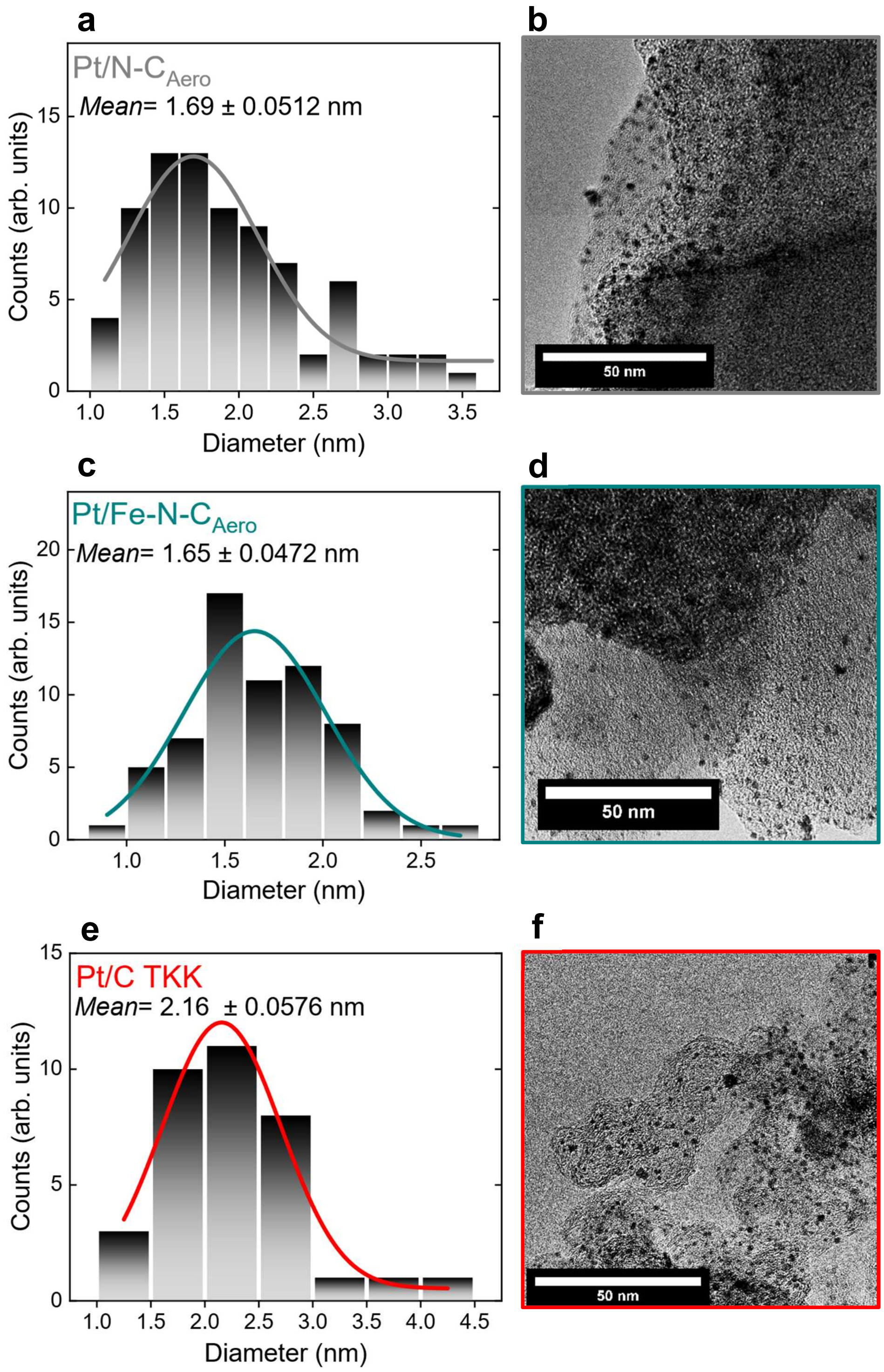

Supplementary Fig. 5 | Pt nanoparticle size distribution in Pt/(Fe–)N–C_{Aero}

Histograms of Pt nanoparticle size distribution estimated from TEM micrographs for **(a-b)** Pt/N–C_{Aero}, **(c-d)** Pt/Fe–N–C_{Aero} and **(e-f)** commercial 10 wt. % Pt/C purchased from TKK. The ImageJ software was used to analyze the TEM images. Only isolated (non-agglomerated) Pt nanoparticles were considered to build the particle size distribution. Source data for **(a) (c) (e)** are provided as a Source Data file.

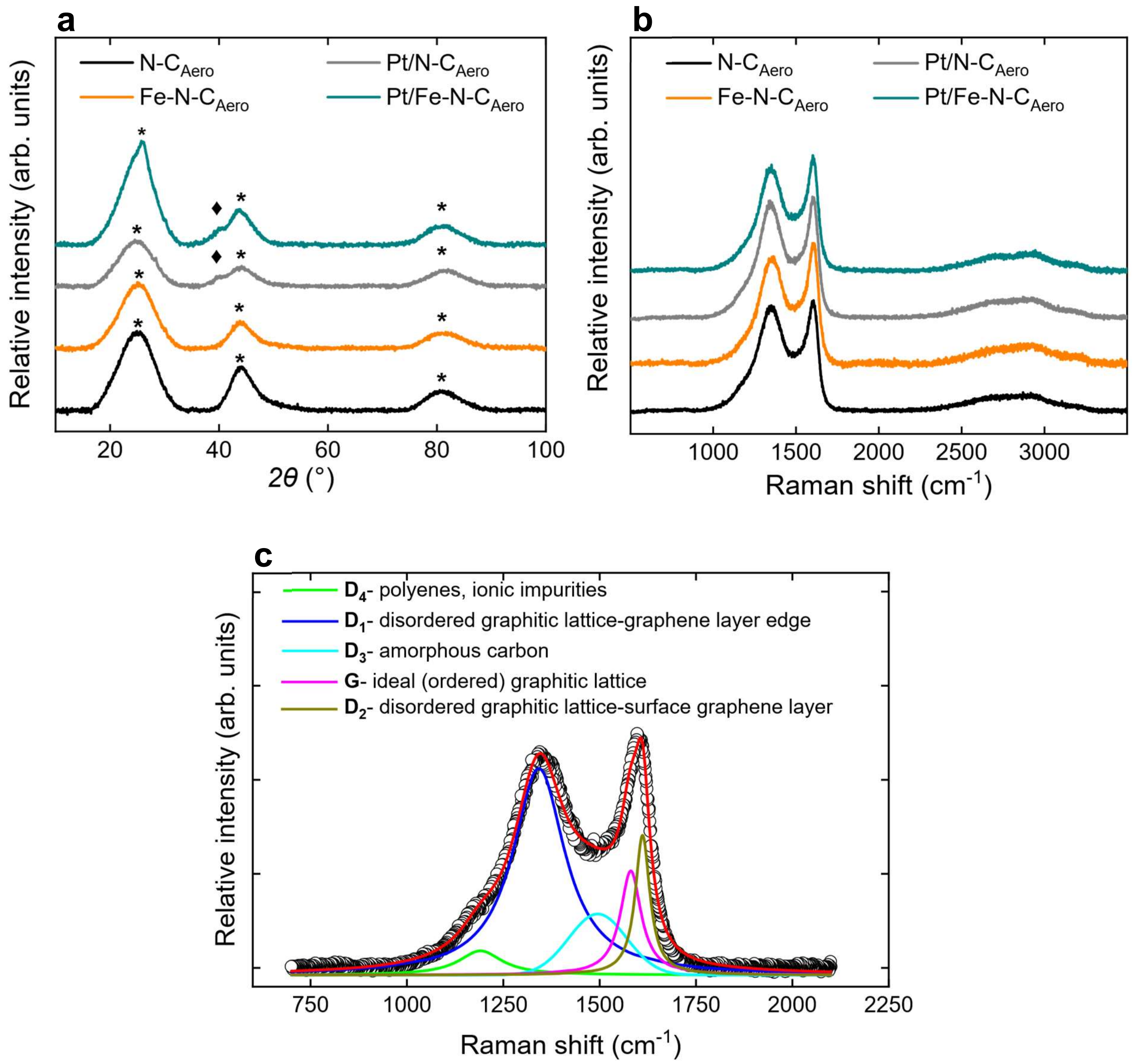

Supplementary Fig. 6 | X-ray diffraction and Raman spectroscopy characterization

(a) X-ray diffractograms and **(b)** Raman spectra for N–C_{Aero}, Fe–N–C_{Aero}, Pt/N–C_{Aero} and Pt/Fe–N–C_{Aero}. **(c)** Example of a Raman spectrum fitting conducted on Fe–N–C_{Aero}. The X-ray diffractograms show the patterns associated with graphite in addition to a small peak at 2θ = 40°, in the samples containing Pt. Two main crystallite phases can be indexed: (*) graphitic C (PDF-2 #41-1487) and (♦) Pt (PDF #27852). The Raman spectra were analyzed using the LLGLL method, which involves fitting with five bands (where '*L*' denotes Lorentzian and 'G' denotes Gaussian profiles).The interested reader is referred to Refs. [8–10] for more details. Source data for are provided as a Source Data file.

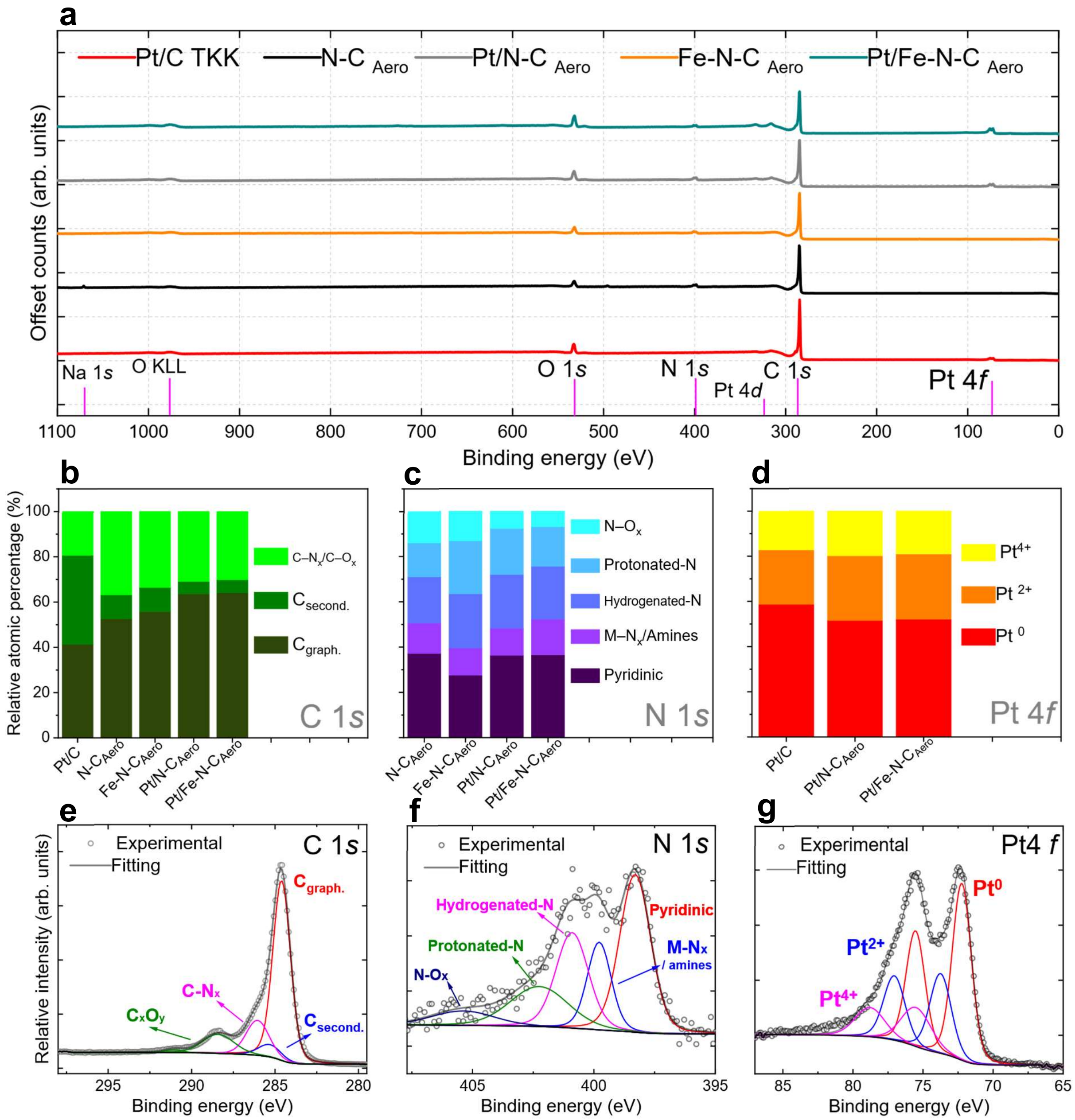

Supplementary Fig. 7 | X-ray photoelectron spectroscopy characterization

(a) Survey X-ray photoelectron spectra of 10 wt. % Pt/C (TKK-TEC-10E10A), N–C_{Aero}, Fe–N–C_{Aero}, Pt/N–C_{Aero} and Pt/Fe–N–C_{Aero} catalysts. Deconvolution method of **(b)** C 1*s*, **(c)** N 1*s* and **(d)** Pt 4*f* regions on the Pt/Fe–N–C_{Aero} catalyst. Relative atomic percentages of different chemical groups found from the fitting of the **(e)** C 1*s*, **(f)** N 1*s* and **(g)** Pt 4*f* narrow regions. Source data are provided as a Source Data file.

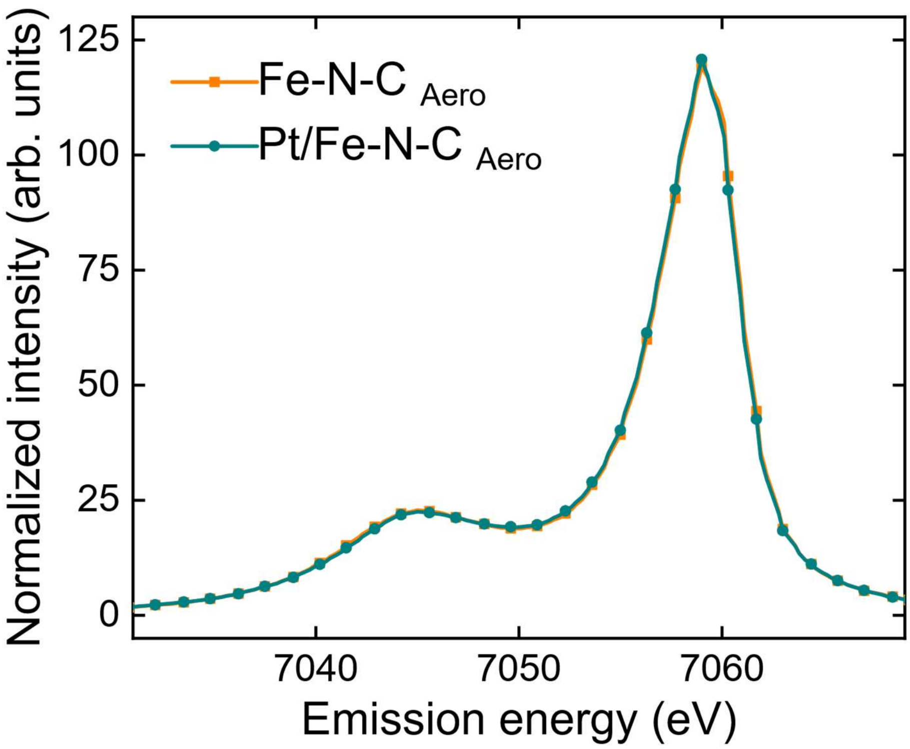

Supplementary Fig. 8 | X-ray emission spectroscopy characterization

*K*β X-ray emission spectra for Fe–N–C_{Aero} and Pt/Fe–N–C_{Aero}. We note that the XANES measurements were conducted using the Fe *K*α line, while the XES measurements were performed using the Fe *K*β line. Although XES can generally be sensitive to oxidation state, the interpretation of oxidation state often relies on *K*α XANES, as *K*β XES is primarily influenced by the spin state.[11] Source data are provided as a Source Data file.

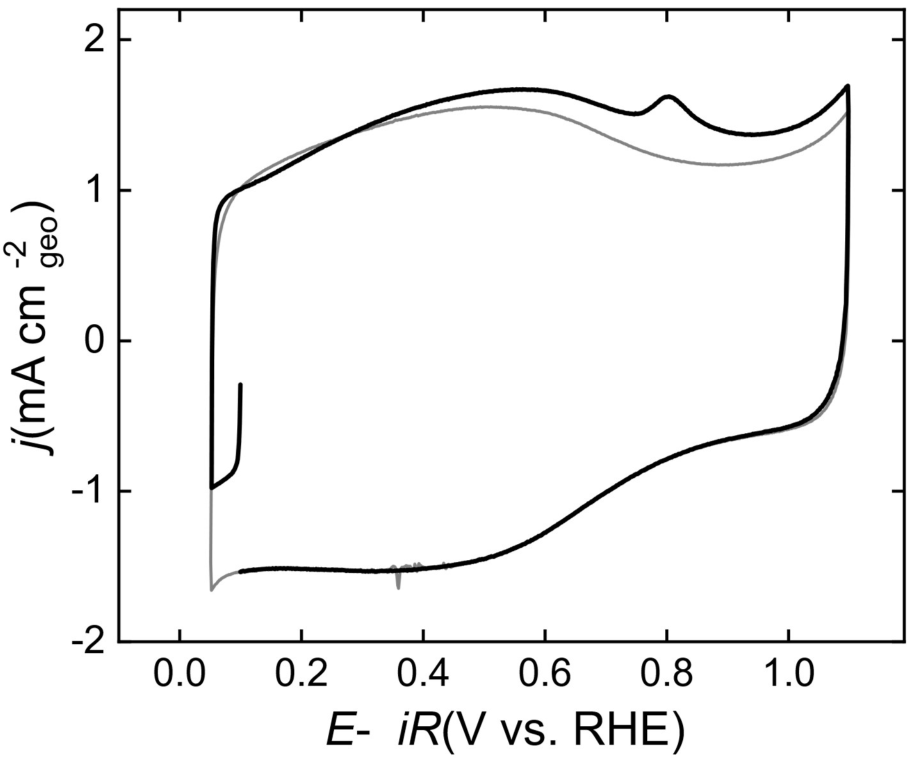

Supplementary Fig. 9 | Electrochemical stripping of adsorbed CO on Pt/Fe–N–C_{Aero}

CO_{ads} stripping curves measured on the Pt/Fe–N–C_{Aero} hybrid catalyst. Ar-saturated 0.1 M H_2SO_4, T = 25 °C, v = 20 mV s^{-1}. The potential was corrected for iR-drop and the measured resistance was *ca*. 18 Ω. Source data are provided as a Source Data file.

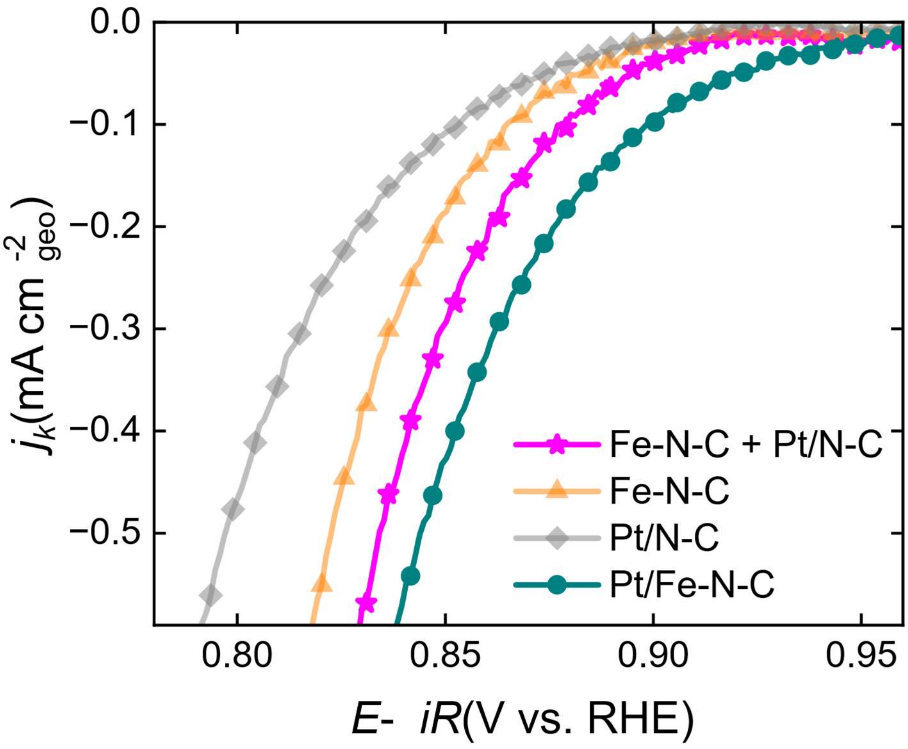

Supplementary Fig. 10 | Comparison of ORR kinetic current density plots

Kinetic current density plot in the low overpotential domain comparing Pt/Fe–N–C_{Aero} and the mathematical sum of Fe–N–C_{Aero} and Pt/N–C_{Aero}. O_2-saturated 0.1 M H_2SO_4, T = 25 °C, v = 2 mV s^{-1}, ω= 1600 rpm. The potential was corrected for iR-drop and the measured resistance was *ca*. 18 Ω. Source data are provided as a Source Data file.

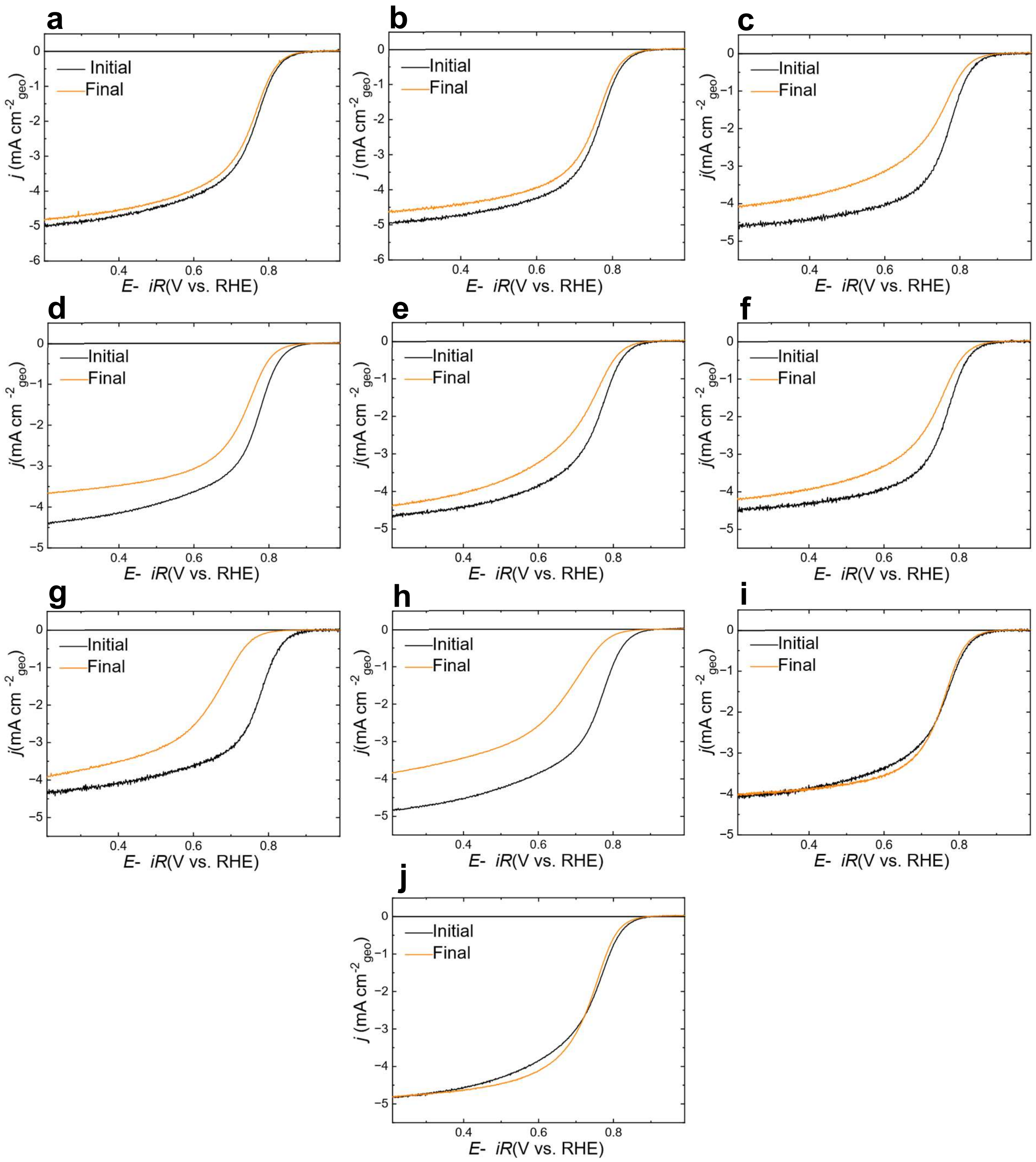

Supplementary Fig. 11 | ORR polarization curves of Fe–N–C$_{Aero}$ before and after each AST

ORR polarization curves performed in O_2-saturated 0.1 M H_2SO_4 electrolyte, recorded at ω = 1600 rpm, T = 25 °C and at v = 2 mV s^{-1} for Fe–N–C$_{Aero}$ before and after **(a)** AST-1, **(b)** AST-2, **(c)** AST-3, **(d)** AST-4, **(e)** AST-5, **(f)** AST-6, **(g)** AST-7, **(h)** AST-8, **(i)** AST-9, and **(j)** AST-10. The potential was corrected for *iR*-drop and the measured resistance was *ca*. 18 Ω. Source data are provided as a Source Data file.

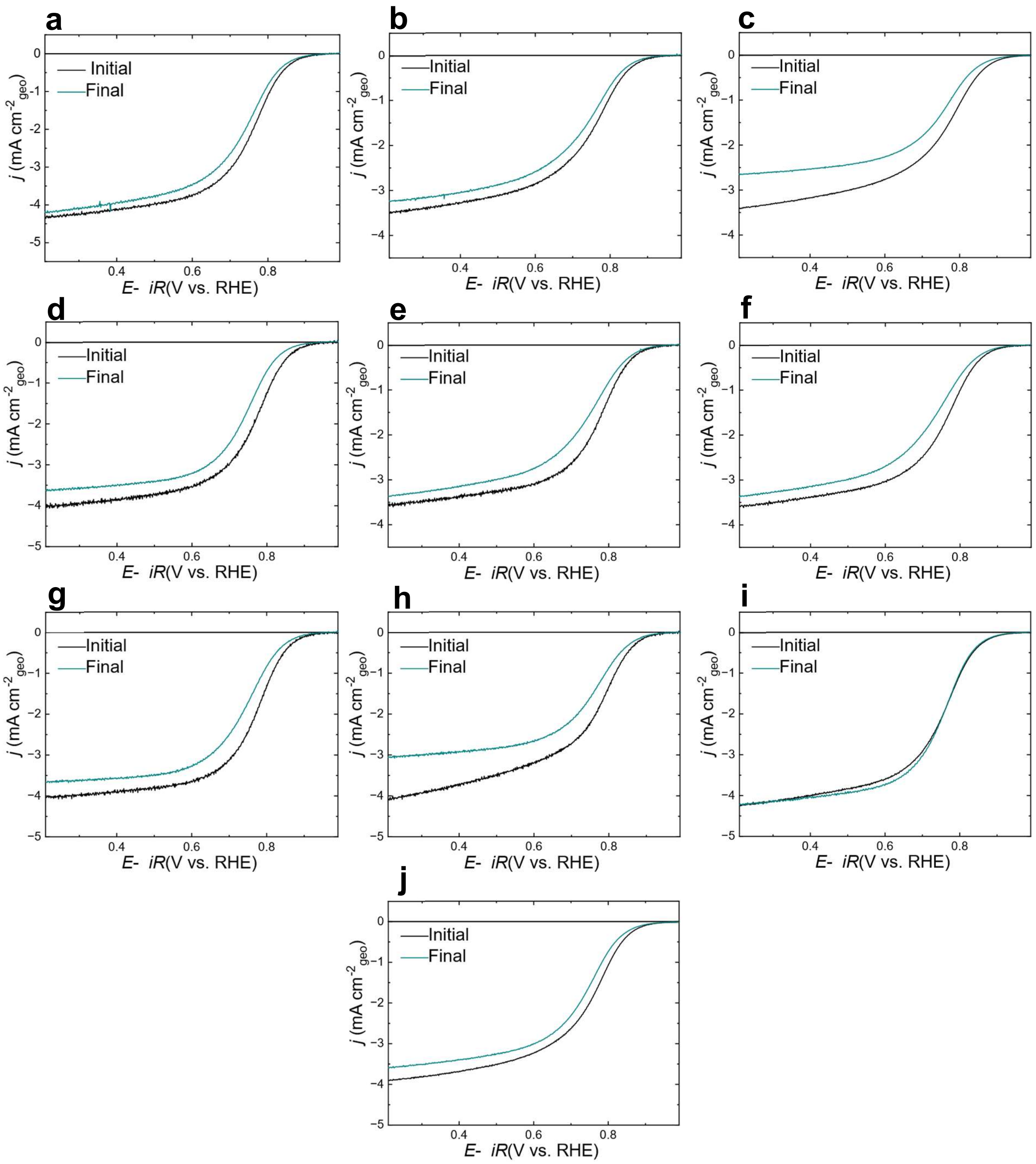

Supplementary Fig. 12 | ORR polarization curves of Pt/Fe–N–C$_{Aero}$ before and after each AST

ORR polarization curves measured in O_2-saturated 0.1 M H_2SO_4 electrolyte, recorded at ω = 1600 rpm, T = 25 °C and at v = 2 mV s^{-1} for Pt/Fe–N–C$_{Aero}$ before and after **(a)** AST-1, **(b)** AST-2, **(c)** AST-3, **(d)** AST-4, **(e)** AST-5, **(f)** AST-6, **(g)** AST-7, **(h)** AST-8, **(i)** AST-9, and **(j)** AST-10. The potential was corrected for iR-drop and the measured resistance was *ca*. 18 Ω. Source data are provided as a Source Data file.

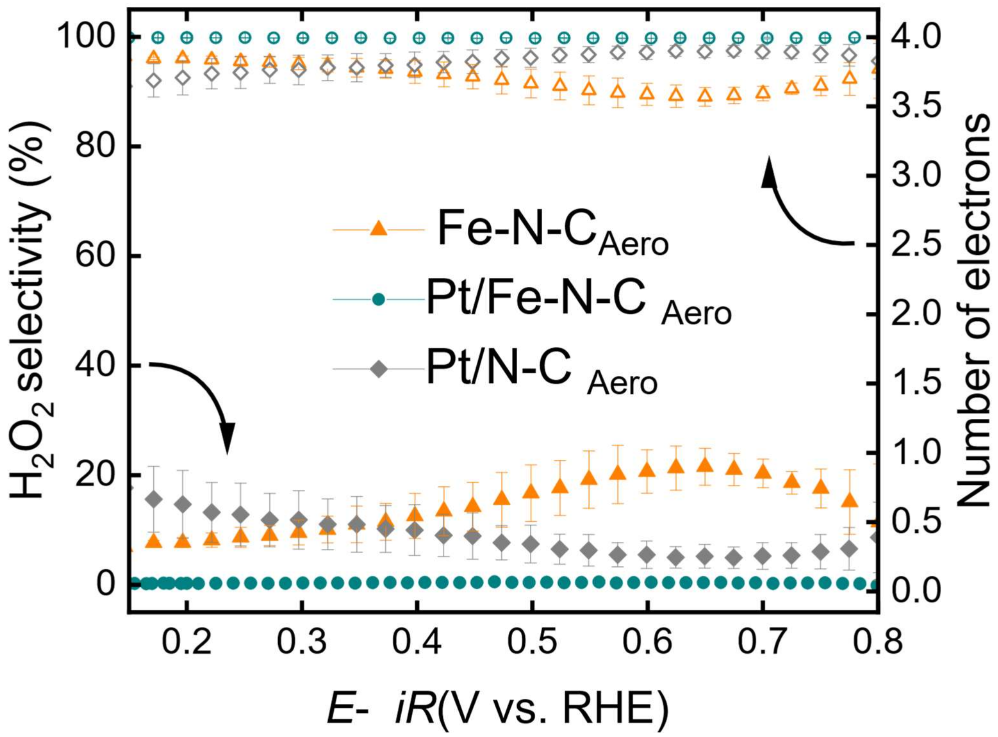

Supplementary Fig. 13 | Effect of Pt on the ORR selectivity

H_2O_2 percentage and number of electrons exchanged during ORR for 100 μg_{powder} $cm^{-2}{}_{geo}$ loading. The error bars are the standard deviation obtained from at least two different measurements. The potential was corrected for *iR*-drop and the measured resistance was *ca*. 18 Ω. Source data are provided as a Source Data file.

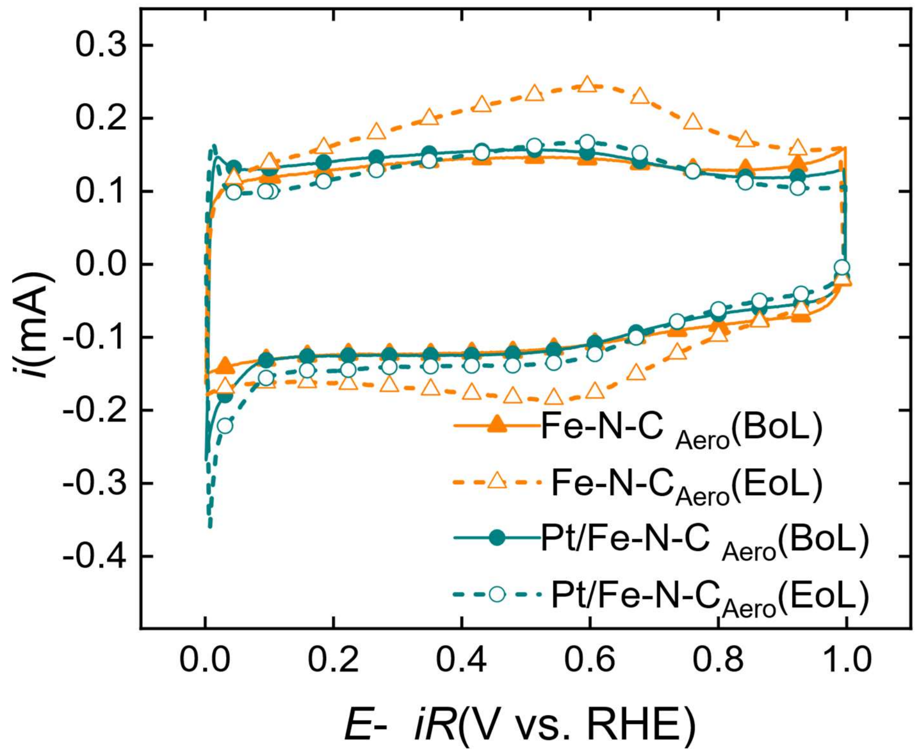

Supplementary Fig. 14 | Comparison of cyclic voltammograms before and after AST-7

Cyclic voltammograms comparing the 'BoL' (solid lines) and 'EoL' (dashed lines) stages of Fe–N–C_{Aero} (orange) and Pt/Fe–N–C_{Aero} (cyan). The measurements were conducted at v = 10 mV s^{-1} and T = 25 °in Ar-saturated 0.1 mol L^{-1} H_2SO_4. The catalyst loading was 400 μg_{powder} cm^{-2}_{geo}. Beginning-of-life ('BoL') denotes the catalyst stage following the break-in, characterization CVs and the ORR polarization curves, *i.e.* prior any AST. End-of-life ('EoL') refers to the final stage, which in this work was chosen to always be after AST-7. The potential was corrected for *iR*-drop and the measured resistance was *ca.* 18 Ω. Source data are provided as a Source Data file.

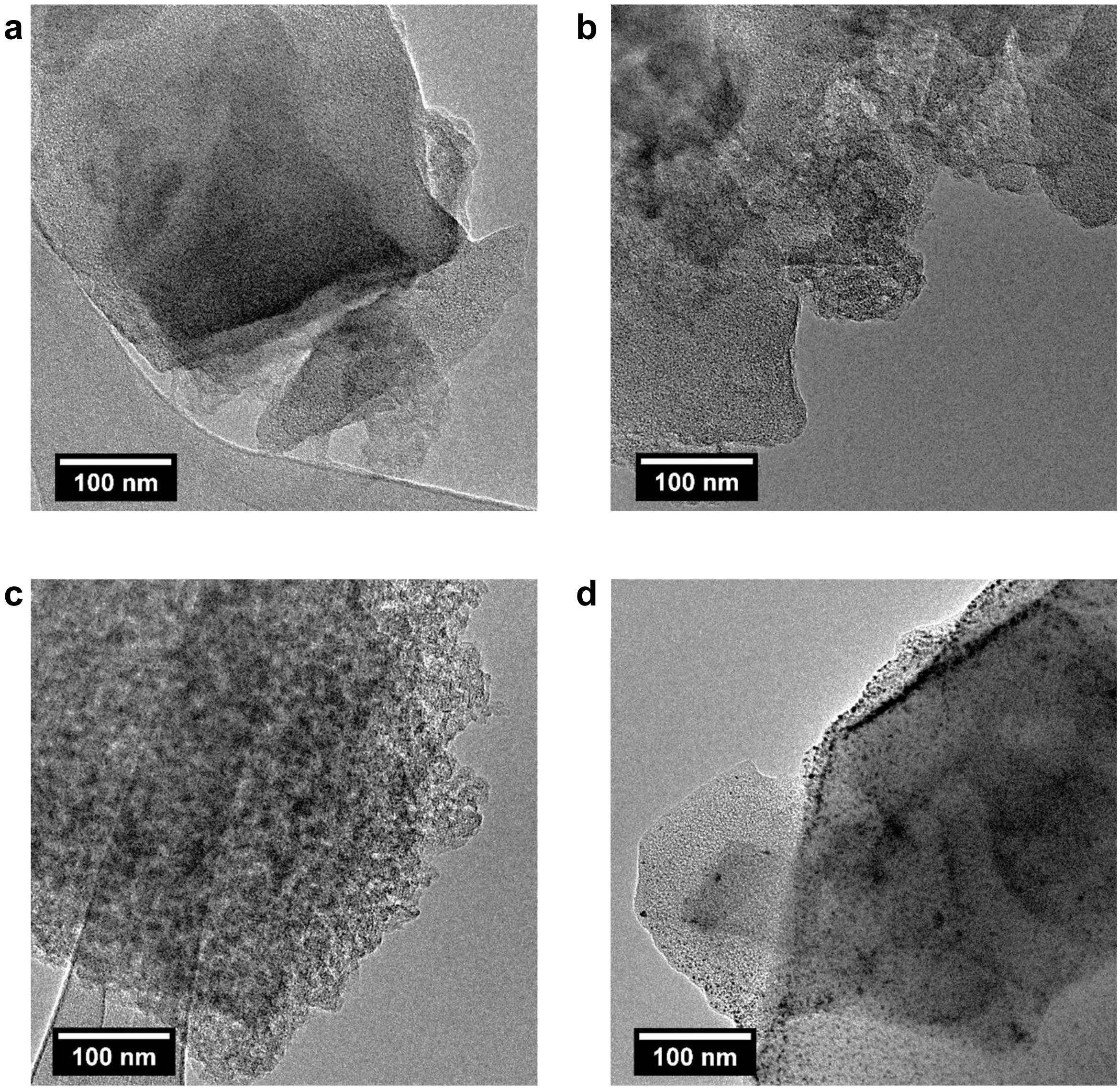

Supplementary Fig. 15 | TEM characterization of catalysts after AST-7

TEM micrographs of Fe–N–C_{Aero} **(a)** after acid exposure, **(b)** at EoL in low-density Fe (LD-Fe) region, **(c)** at EoL in high-density Fe (HD-Fe) region, and **(d)** Pt/Fe–N–C_{Aero} at EoL. End-of-life ('EoL') refers to the final stage, which in this work was chosen to always be after AST-7.

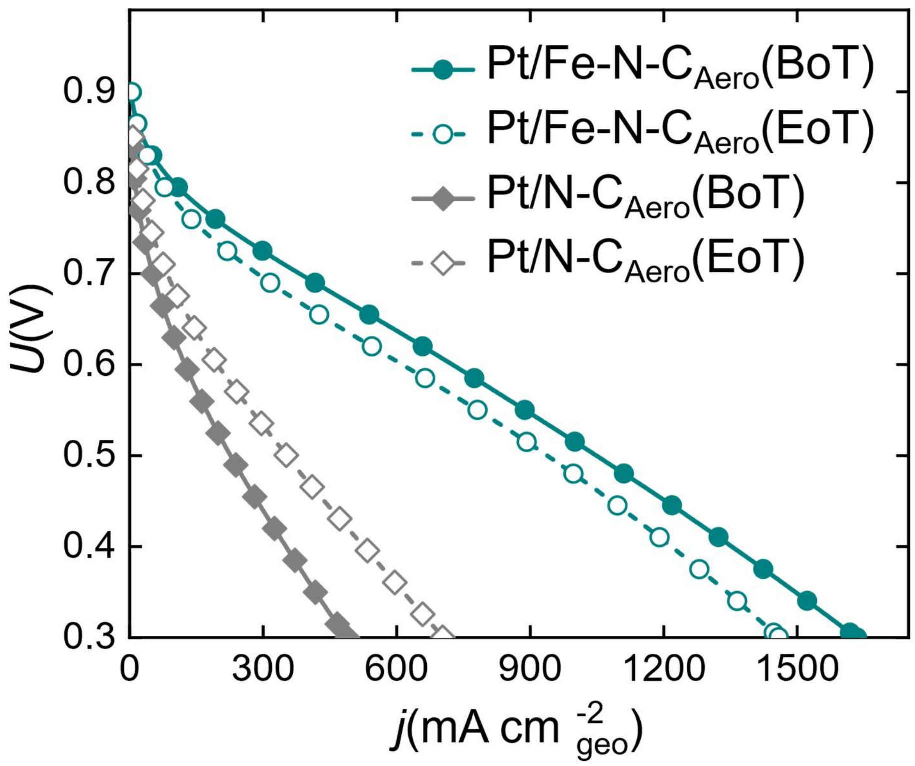

Supplementary Fig. 16 | BoT and EoT PEMFC polarization curves of Pt/(Fe–)N–C_{Aero}

Polarization curves in PEMFC device were obtained at T = 80 °C comparing BoT and end-of-test (EoT, at U = 0.5 V for 20 h) of Pt/Fe–N–C_{Aero} and Pt/N–C_{Aero}. All PEMFC voltages and polarization curves are uncorrected for iR-drop. Source data are provided as a Source Data file.

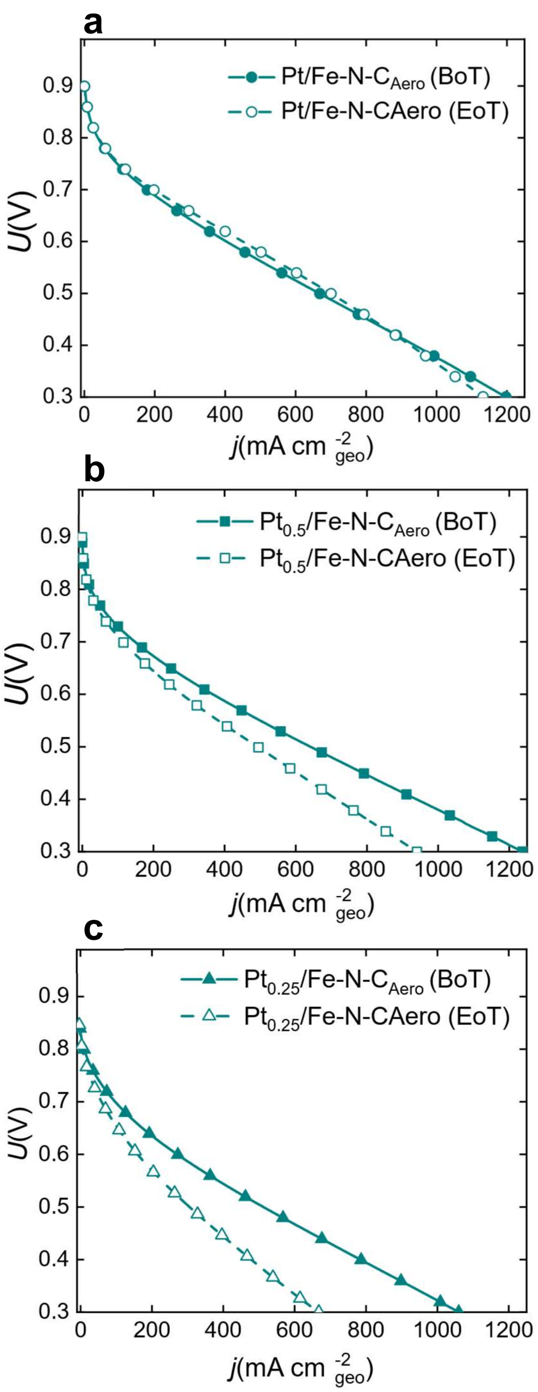

Supplementary Fig. 17 | Effect of Pt content on BoT and EoT PEMFC polarization curves of platinized Fe–N–C$_{Aero}$

Polarization curves in PEMFC device were obtained at T = 80 °C comparing BoT and end-of-test (EoT, at U = 0.5 V for 50 h) of **(a)** Pt/Fe–N–C$_{Aero}$, **(b)** Pt$_{0.50}$/Fe–N–C$_{Aero}$ and **(c)** Pt$_{0.25}$/Fe–N–C$_{Aero}$, comprising 1.0, 0.50 and 0.25 wt. % Pt, respectively Note that the BoT performance of Pt/Fe–N–C$_{Aero}$ here is slightly lower than in all other graphs because the Pt deposition to reach 1.0, 0.50 and 0.25 wt. % Pt was made at the same time to avoid any biases, and the Fe–N–C$_{Aero}$ batch had aged slightly during *ca.* 9 months shelf storage in air relative to all other PEMFC experiments reporting Fe–N–C$_{Aero}$ or Pt/Fe–N–C$_{Aero}$ data. All PEMFC voltages and polarization curves are uncorrected for *iR*-drop. Source data are provided as a Source Data file.

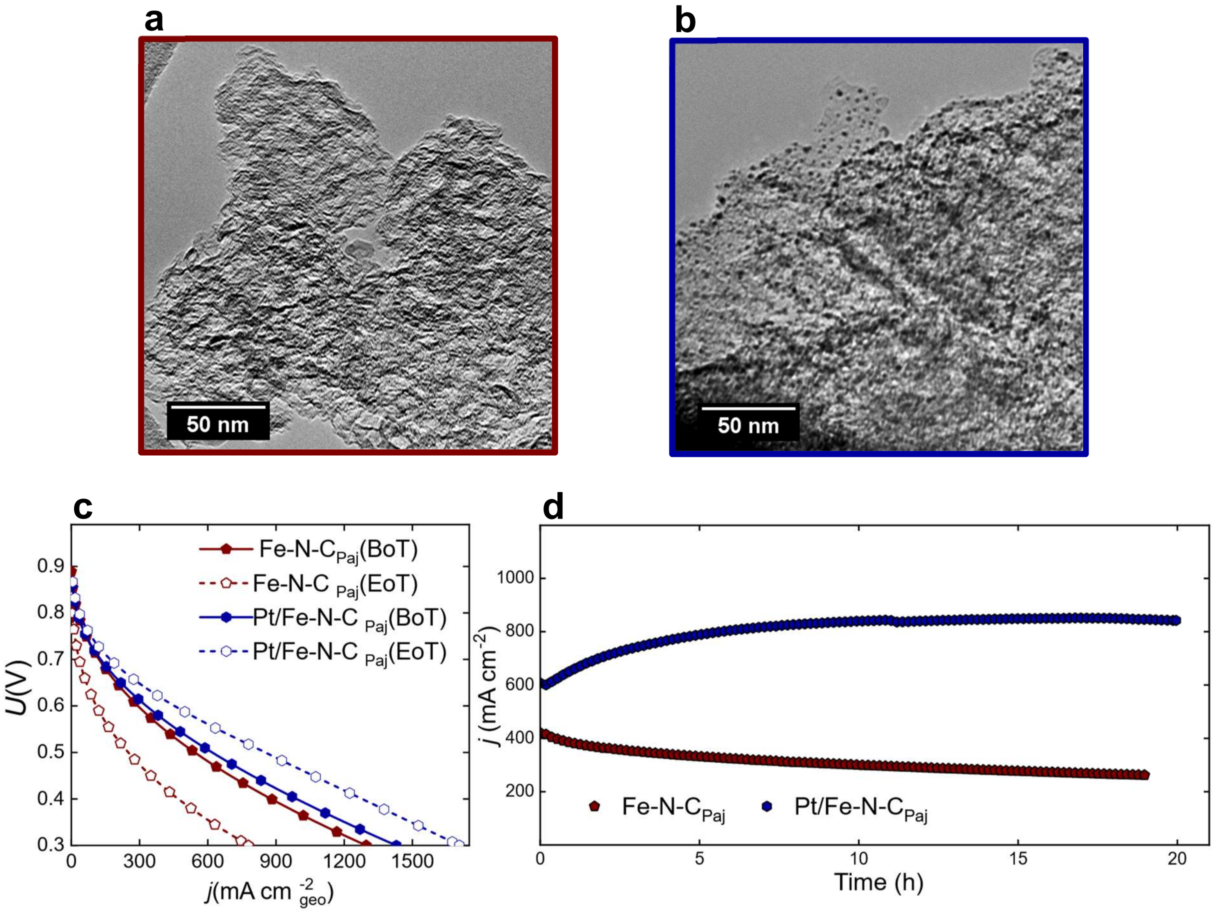

Supplementary Fig. 18 | Stabilization by Pt of a Fe–N–C catalyst from Pajarito Powder

TEM images of **(a)** Fe–N–C_{Paj} and **(b)** Pt/Fe–N–C_{Paj}. PEMFC experiments were operated at T = 80 °C comparing Fe–N–C_{Paj} and Pt/Fe–N–C_{Paj} **(c)** Polarization curves obtained at the BoT and EoT, and **(d)** chronoamperometries measured at U = 0.5 V. The average Pt particle size estimated for Pt/Fe–N–C_{Paj} from **(b)** was 1.96 ± 0.0973 nm. All PEMFC voltages and polarization curves are uncorrected for *iR*-drop. Source data for **(c)** **(d)** are provided as a Source Data file.

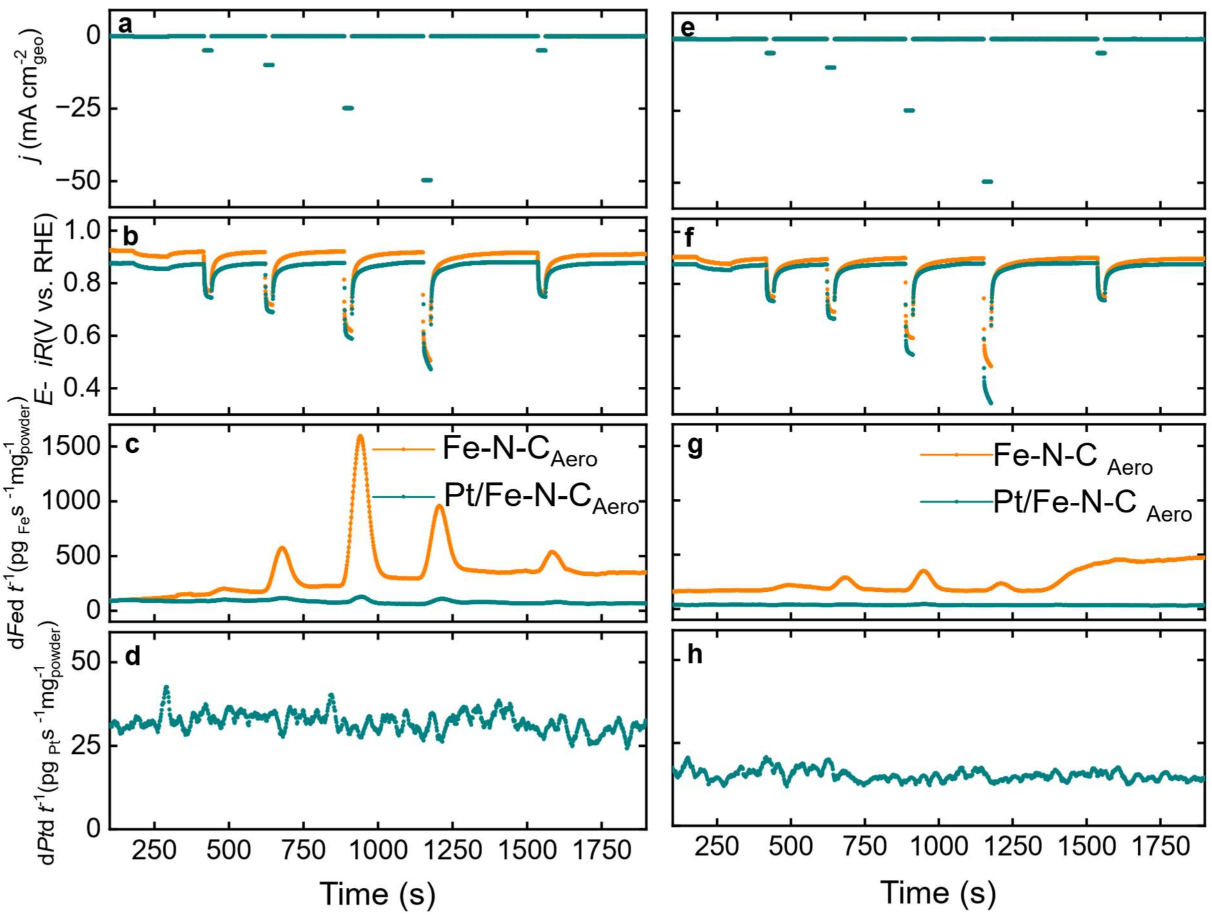

Supplementary Fig. 19 | Online measurement of iron leaching rate of (Pt/)Fe–N–C$_{Aero}$

S-GDE-ICP-MS measurements of Fe–N–C$_{Aero}$ and Pt/Fe–N–C$_{Aero}$ during activity test pre (left) and post (right) 200 galvanostatic cycles AST protocol conducted under room temperature in an O_2-saturated 0.1 M $HClO_4$ at a flow rate of 50 mL min^{-1}. Current density **(a, e)**, potential **(b, f)**, and Fe **(c, g)** and Pt **(d, h)** dissolution signals normalized to catalysts loading of 0.93 mg$_{powder}$ cm$^{-2}_{geo}$ and 1.03 mg$_{powder}$ cm^{-2} for Fe–N–C$_{Aero}$ and Pt/Fe–N–C$_{Aero}$, respectively. The *iR*-drop was 100 % post corrected for each current density. Source data are provided as a Source Data file.

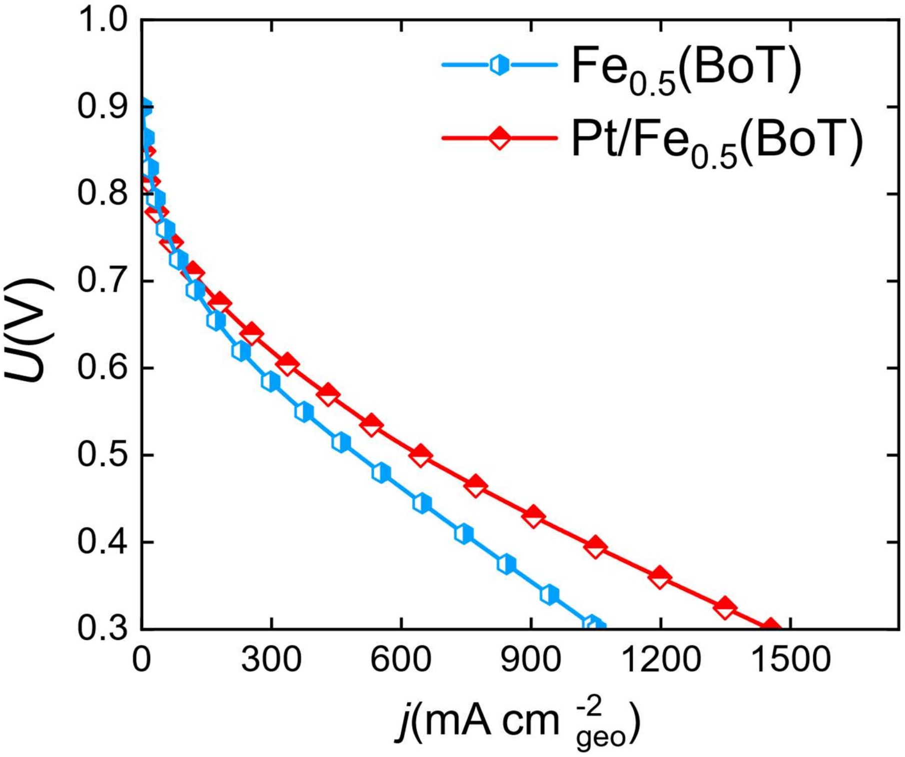

Supplementary Fig. 20 | BoT PEMFC polarization curves of (Pt/)$Fe_{0.5}$

BoT polarization curves for Pt/$Fe_{0.5}$ and $Fe_{0.5}$, with the $Fe_{0.5}$ batch being the same as used for preparing the platinized Pt/$Fe_{0.5}$, and cathode and MEA preparation performed identically as well. All PEMFC voltages and polarization curves are uncorrected for *iR*-drop. Source data are provided as a Source Data file.

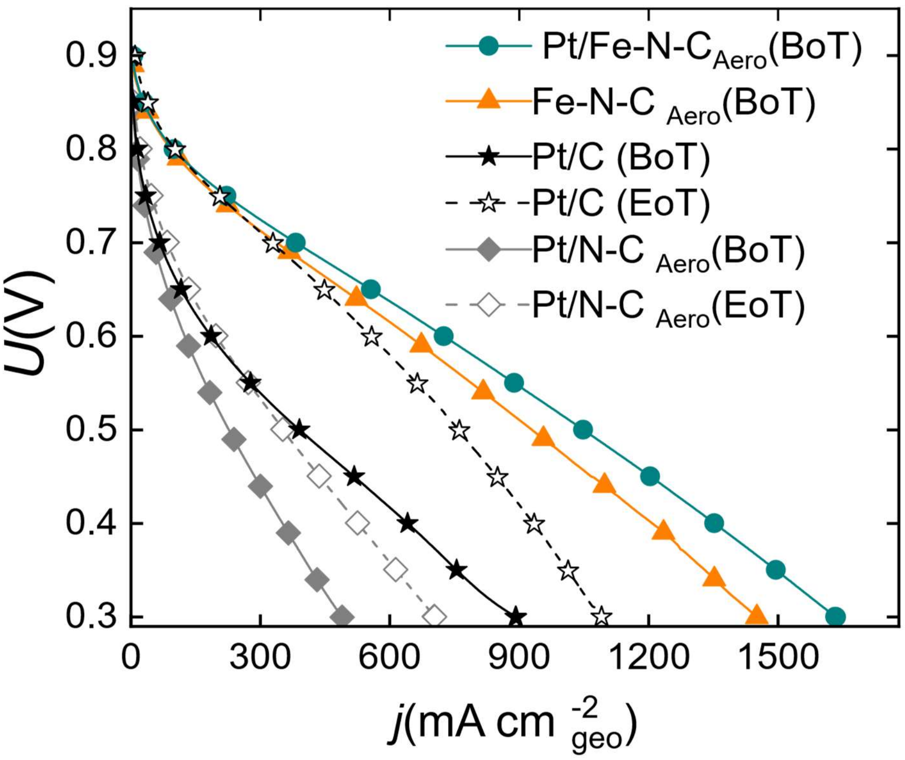

Supplementary Fig. 21 | Comparison of BoT and EoT PEMFC polarization curves for various cathode layers at 40 μg$_{Pt}$ cm$^{-2}_{geo}$

BoT polarization curves for Fe–N–C$_{Aero}$, Pt/Fe–N–C$_{Aero}$, Pt/N–C$_{Aero}$, Pt/C, as well as EoT curves for Pt/N–C$_{Aero}$ and Pt/C. The total cathode catalyst loading was 4 mg$_{powder}$ cm$^{-2}_{geo}$ for Fe–N–C$_{Aero}$, Pt/Fe–N–C$_{Aero}$ and Pt/N–C$_{Aero}$ (resulting in *ca*. 40 μg$_{Pt}$ cm$^{-2}_{geo}$ at the cathode for Pt/Fe–N–C$_{Aero}$ and Pt/N–C$_{Aero}$) while the total cathode catalyst loading for the commercial Pt/C (40 wt. % Pt on Vulcan XC72) was adjusted to result in 40 μg$_{Pt}$ cm$^{-2}_{geo}$ at the cathode. The cathode ink deposition method and MEA preparation were otherwise performed identically. See Supplementary Note 5 for a discussion of the figure results. All PEMFC voltages and polarization curves are uncorrected for *iR*-drop. Source data are provided as a Source Data file.

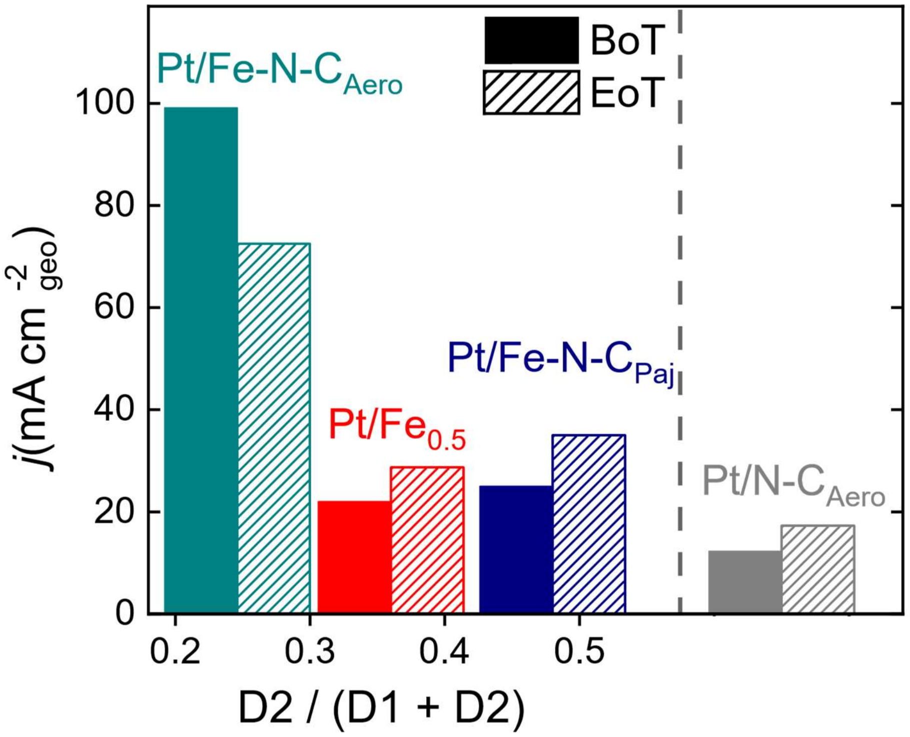

Supplementary Fig. 22. | Correlation between ORR activity change in PEMFC and the relative amount of D2 type of FeN_4 sites

Change in current density at 0.8 V (uncorrected for *iR*-drop) in PEMFC from BoT to EoT as a function of the D2 / (D1 + D2) ratio derived from ^{57}Fe Mössbauer spectroscopy of the parent Fe–N–C materials. The change in current density is also shown for Pt/N–C_{Aero} as a comparison to Pt/Fe–N–C_{Aero}. Source data are provided as a Source Data file.

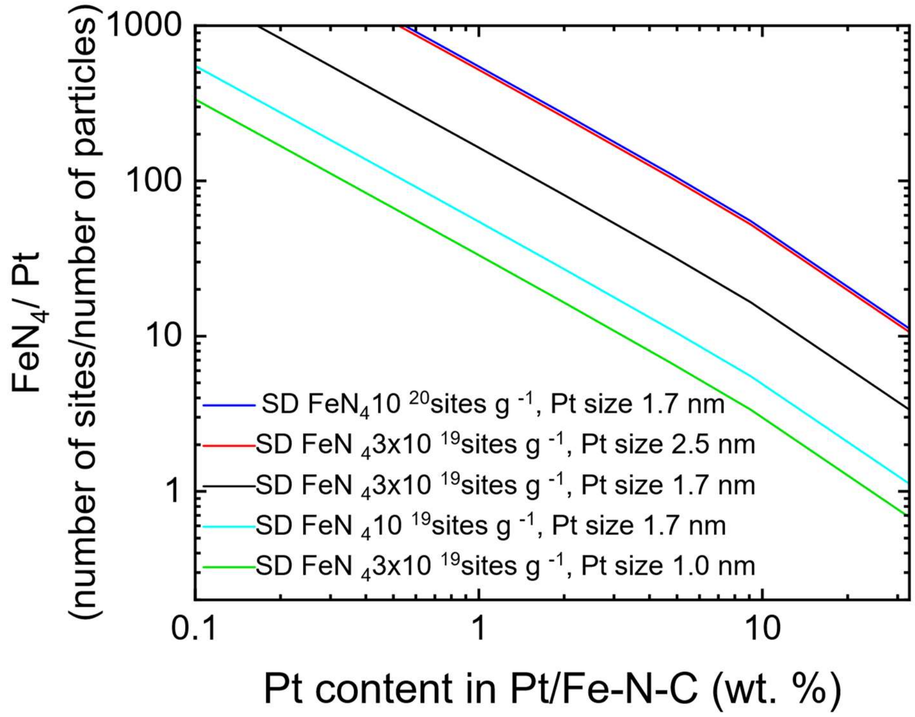

Supplementary Fig. 23 | Ratio of number of FeN_4 sites to number of Pt particle

Calculated ratio of the number of FeN_4 sites to the number of Pt particles, as a function of the Pt content in Pt/Fe–N–C hybrid materials. The different SD values and Pt particle size values used for the calculations are shown in the legend. See Supplementary Note 6 for the details on how the calculations were performed. Source data are provided as a Source Data file.

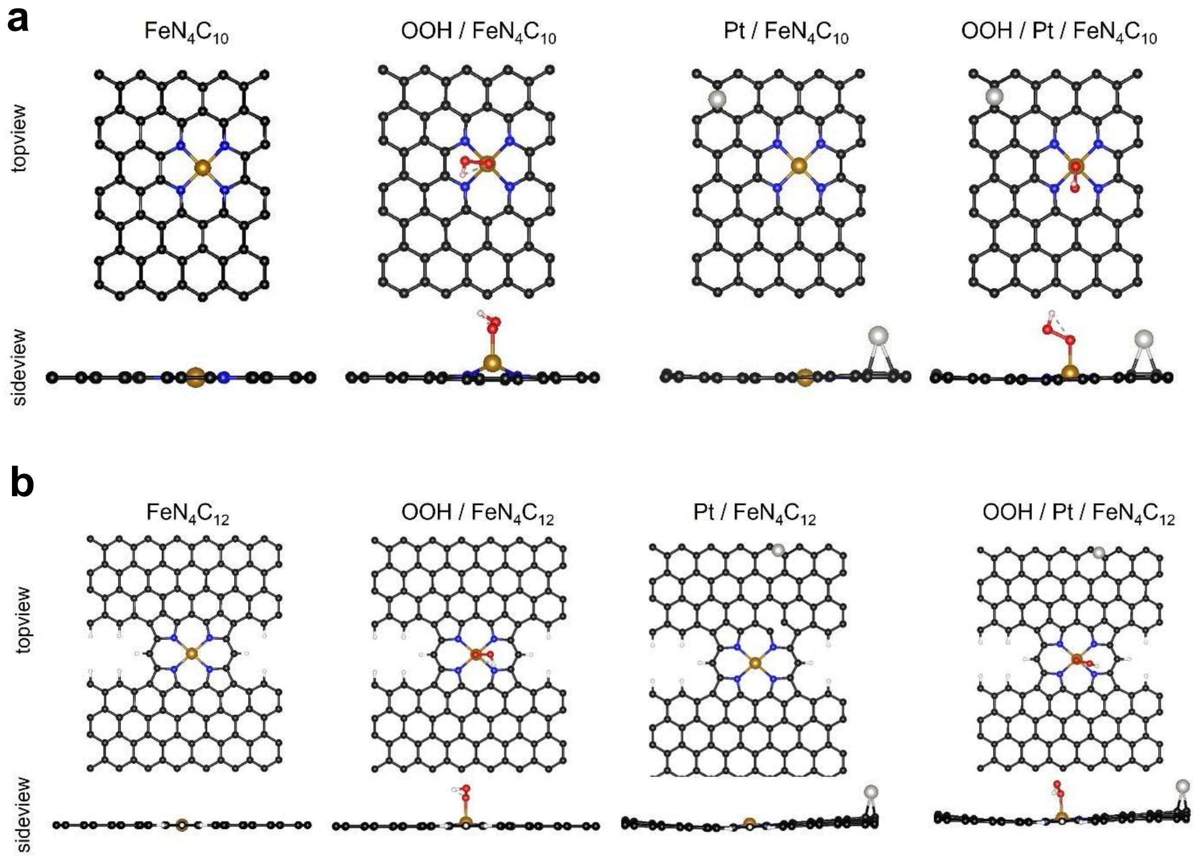

Supplementary Fig. 24 | DFT optimized cells of the Pt/Fe–N–C model systems

DFT optimized unit cells of ferric and ferrous **(a)** FeN_4C_{10} and **(b)** FeN_4C_{12} models without and with adsorbed Pt atom on graphene. OOH* is adsorbed on Fe in the second and fourth structures in **(a)** and **(b)**. The atoms' color code is the following: carbon = black; iron = brown; platinum = grey; nitrogen = blue; hydrogen = white; oxygen = red. The total spin was $S_{tot.} = 1$ without OOH and $S_{tot.} = 3/2$ with OOH.

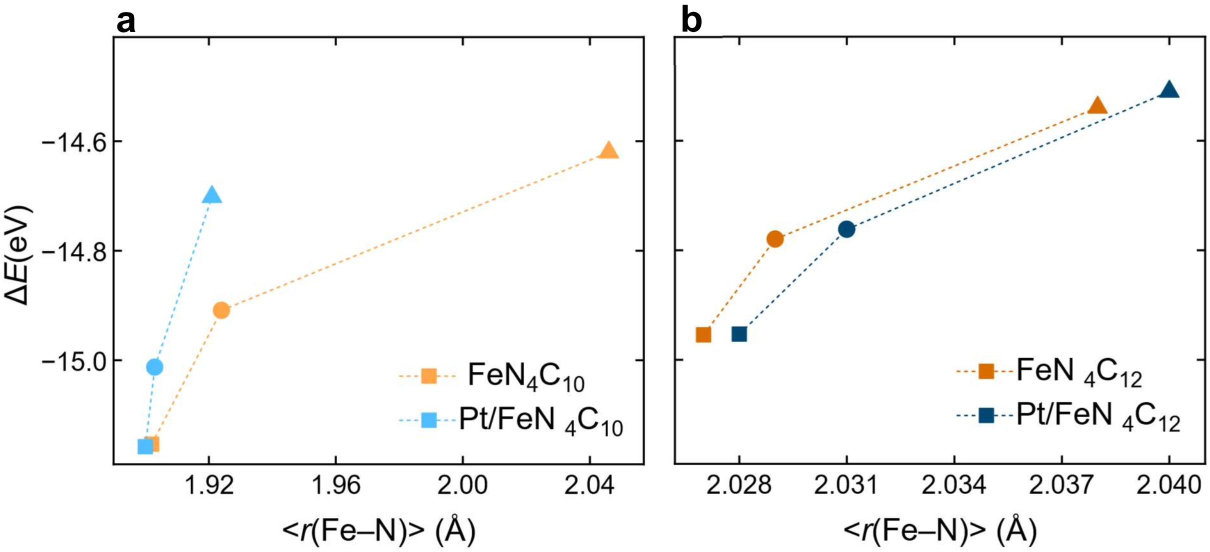

Supplementary Fig. 25 | Calculated OOH-Fe interaction energies

OOH*-Fe interaction energies, computed from Eq. 5 in Methods, in **(a)** FeN_4C_{10} and **(b)** FeN_4C_{12} models without and (light and dark orange) with adsorbed Pt atom (light and dark blue). The total spin imposed ($S_{tot.}$) are represented in each point by squares ($S_{tot.}$ = 1/2), circles ($S_{tot.}$ = 3/2), and triangles ($S_{tot.}$ = 5/2). The x-axis reports the average Fe-N distance for the OOH/(Pt)/FeN_4C_x models.

For a given imposed total spin, the Pt adsorption leads to slightly increased interaction of Fe with OOH^* (by a maximum of 0.1 eV) in the case of the FeN_4C_{10} model (Supplementary Fig. 24a, more negative ΔE for Pt/FeN_4C_{10} *vs*. FeN_4C_{10} when compared at a same total spin), but slightly decreased interaction (by a maximum of 0.05 eV) in the case of the FeN_4C_{12} model (Supplementary Fig. 24b). The figure also visualizes the shortening of the Fe-N distance upon Pt adsorption (FeN_4C_{10} model) or the small increase in Fe-N distance upon Pt adsorption (FeN_4C_{12} model). Source data are provided as a Source Data file.

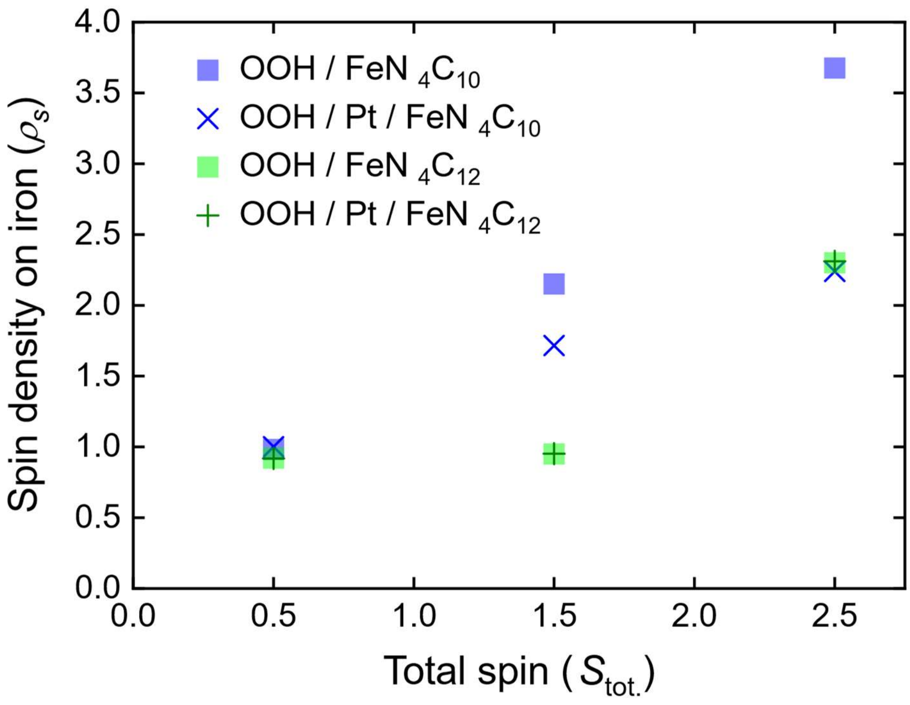

Supplementary Fig. 26 | Calculated Spin-density on iron as a function of the total spin

Spin-density on iron, ρ_s, as a function of the total spin, $S_{tot.}$, imposed on the periodic structures. The figure corresponds to calculations performed with OOH adsorbed atop the Fe center. The spin density is defined as the difference between the spin up and spin down densities, $\rho_s = \rho_s(\alpha) - \rho_s(\beta)$, where α denotes spin-up and β denotes spin-down densities. Source data are provided as a Source Data file.

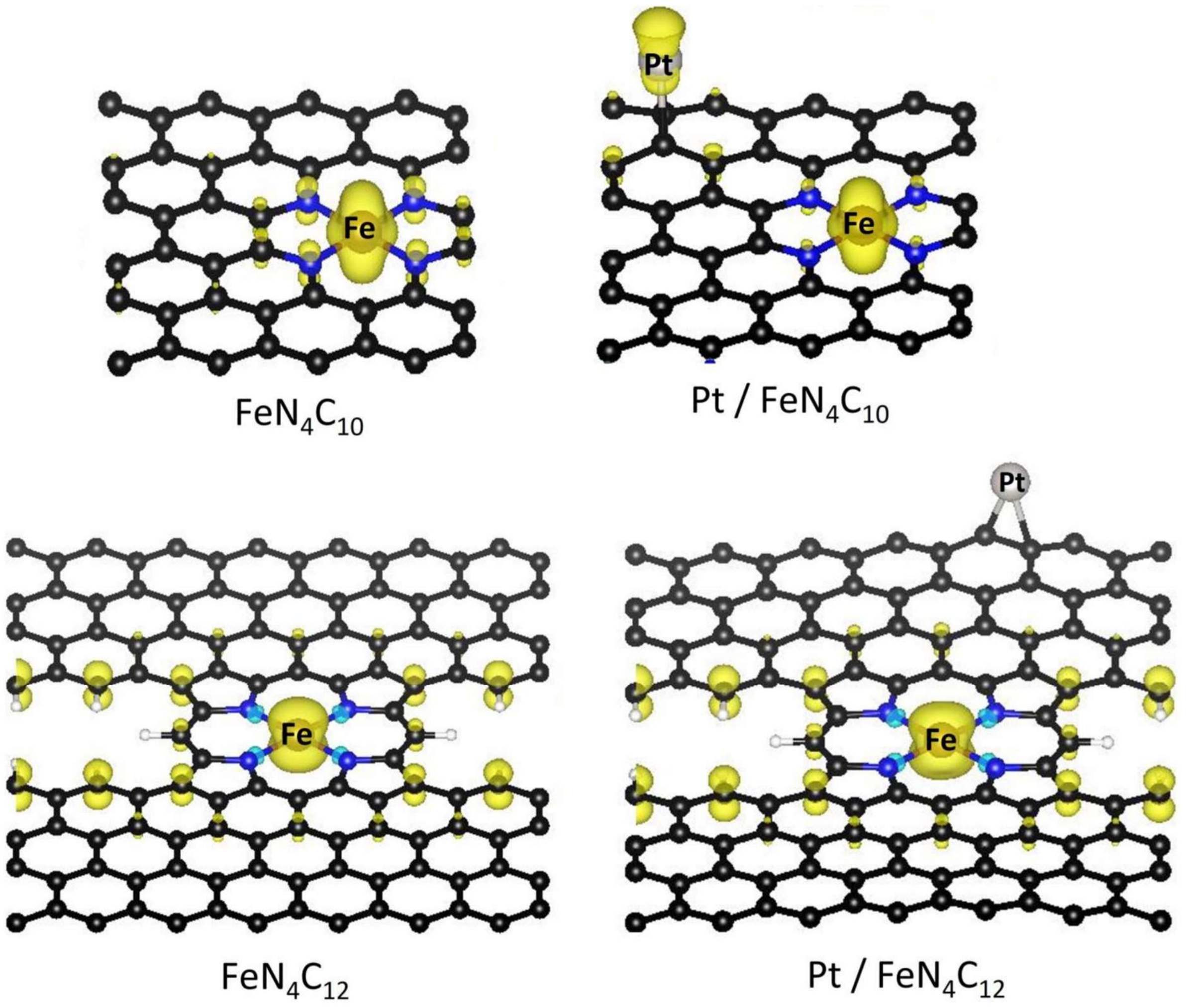

Supplementary Fig. 27 | Calculated atomic spin distribution

Atomic spin distribution in Pt / FeN_4C_x models obtained when imposing $S_{tot.} = 2$. The color code is the following: yellow for spin-up and cyan for spin-down.

Supplementary Information Tables

Supplementary Table 1 | Porous structure of the Fe–N–C_{Aero}, Fe–N–C_{Paj} (batch PMF-D14401, Pajarito Powder) and $Fe_{0.5}$ powders. Specific surface area (BET), mesopore volume derived from BJH analysis of the adsorption isotherms, total pore volume and micropore volume (the latter was derived from the total pore volume minus the pore volume assessed by BJH analysis for all pores of size > 2 nm):

	**BET area ($m^2 g^{-1}$)**	**Microporous volume ($cm^3 g^{-1}$)**	**Mesoporous volume ($cm^3 g^{-1}$)**	**Total pore volume ($cm^3 g^{-1}$)**
**Fe–N-C_{Aero}**	1191	0.46	0.02	0.51
**Fe-N-C_{Paj}**	755	0.25	0.85	1.28
**$Fe_{0.5}$**	455	0.19	0.04	0.25

Supplementary Table 2 | Parameters derived from the fittings of Mössbauer spectra measured at -268 °C of the Fe–N–C_{Aero}, Fe–N–C_{Paj} (batch PMF-D14401, Pajarito Powder) and $Fe_{0.5}$ powders: relative area (*RA* – %), isomer shift (*IS* – $mm \cdot s^{-1}$), quadrupole splitting (*QS* – $mm \cdot s^{-1}$), linewidth (*LW* – $mm \cdot s^{-1}$) and hyperfine field (*H* – Tesla) of each component.

(**): batch from 2021 and same batch was used to acquire the BoT and EoT polarization curves shown in Fig. 6c as well as BoT and EoT Mössbauer spectrum of $Fe_{0.5}$ cathode shown in Figs. 6a,d. These data were already published in Ref.[2].

	**Component**	***RA*** **%**	***IS*** **$mm \cdot s^{-1}$**	***QS*** **$mm \cdot s^{-1}$**	***LW*** **$mm \cdot s^{-1}$**	***H*** **Tesla**	**Assignment**
**Fe-N-C_{Aero}**	D1	61	0.47	0.97	0.91	-	HS O-$Fe^{3+}N_4$
	D2	20	0.47	2.35	0.89	-	LS or MS $Fe^{2+}N_4$
	Sextet 1	19	0.30	-	1.27	25.9	Fe_3C
**Fe-N-C_{Paj}**	D1	52	0.52	0.94	1.03	-	HS O-$Fe^{3+}N_4$
	D2	48	0.72	2.59	1.61	-	LS or MS $Fe^{2+}N_4$
**$Fe_{0.5}$ (**)**	D1	64	0.34	0.94	0.65	-	HS O-$Fe^{3+}N_4$
	D2	36	0.36	2.59	1.40	-	LS or MS $Fe^{2+}N_4$

Supplementary Table 3 | Best-fit parameters obtained from the Fe *K*-edge EXAFS analysis of Fe–N–C_{Aero} and Pt/Fe–N–C_{Aero}. *R* is the bond distance, σ^2 is the Debye-Waller factor, and CN is the coordination number.

	**Fe–N–C_{Aero}**			**Pt/Fe–N–C_{Aero}**		
	R(Å)	σ^2(10^{-3} Å^2)	CN	*R*(Å)	σ^2(10^{-3} Å^2)	CN
**Fe-N**	2.05 ± 0.02	7.9 ± 0.5	3.8 ± 0.3	2.05 ± 0.01	5.6 ± 0.7	3.5 ± 0.1
**Fe-O**	1.91 ± 0.03	5.6 ± 0.9	1.8 ± 0.2	1.88 ± 0.02	6.7 ± 1.0	2.0 ± 0.1
**Fe-C**	3.61 ± 0.04	30 ± 5	10 ± 1	3.46 ± 0.03	30 ± 5	8.6 ± 0.3

Supplementary Table 4 | Relative atomic percentages of different chemical groups obtained by peak-fitting of the C 1*s* regions of the X-ray photoelectron spectra recorded on Pt/C TKK, N–C_{Aero}, Fe–N–C_{Aero}, Pt/N–C_{Aero} and Pt/Fe–N–C_{Aero}. Four contributions were considered to fit the C 1*s* region: graphitic carbons (*sp*^{2}), secondary carbons (C*-C-O_x/C*-C-N_x), C-N_x and C-O_x (C-OH, C-OC, C=O, COOH, shake-up from aromatic structure) groups, corresponding to 284.8 eV, 285.3 – 285.6 eV, 286.1 – 286.3 eV, > 287 eV binding energies, respectively, agreeing with the literature.[12–14]

Catalysts	Info	$C_{graph.}$	$C_{second.}$	CN_x/C-O_x	C-O_x	C-O_x	C-O_x	C-O_x
**Pt/C - TKK**	**Peak (eV)**	284.8	285.6	287.0	290.6	289.2	290.8	292.9
	**Area (%)**	41.4	39.3	9.9	0.8	8.5	0.2	0.0
**N-C_{Aero}**	**Peak (eV)**	284.8	285.3	286.1	287.4	289.3	291.0	292.8
	**Area (%)**	52.6	10.6	15.4	9.7	5.6	3.4	2.7
**Fe-N-C_{Aero}**	**Peak (eV)**	284.8	285.3	286.1	287.5	298.4	291.3	293.4
	**Area (%)**	55.8	10.7	14.1	9.0	5.7	3.5	1.2
**Pt/N-C_{Aero}**	**Peak (eV)**	284.8	285.8	286.2	288.3	290.8	290.4	292.4
	**Area (%)**	63.6	5.6	13.9	9.0	0.6	7.2	0.2
**Pt/Fe-N-C_{Aero}**	**Peak (eV)**	284.8	285.5	286.3	288.6	291.3	291.8	293.4
	**Area (%)**	54.1	5.8	16.2	12.2	1.0	0.6	0.0

Supplementary Table 5 | Relative atomic percentages of different chemical groups obtained by peak-fitting of the N 1*s* regions of the X-ray photoelectron spectra recorded on N–C_{Aero}, Fe–N–C_{Aero}, Pt/N–C_{Aero} and Pt/Fe–N–C_{Aero}. Five contributions were considered to fit the N 1*s* region: pyridinic, M-N_x and/or amines, hydrogenated-N (pyrrolic, hydrogenated-pyridine), protonated-N (quaternary nitrogen, protonated pyridine) and oxidized (N-OH, N=O), corresponding to 398.3 – 398.8 eV, 399.4 – 400.0 eV, 401.5 – 403.5 eV and > 403 eV binding energies, respectively, following the literature values.[12,13]

Catalysts	Info	Pyridinic	M-N_x/ amines	Hydrogenated-N	Protonated-N	N-O_x	N-O_x
N-C_{Aero}	Peak (eV)	398.3	399.7	400.9	401.6	406.4	403.7
	Area (%)	37.2	13.4	20.5	15.0	4.4	9.6
Fe-N-C_{Aero}	Peak (eV)	398.4	399.5	400.9	401.6	404.0	406.5
	Area (%)	27.6	12.0	24.0	23.4	7.8	5.3
Pt/N-C_{Aero}	Peak (eV)	398.4	399.7	400.9	401.7	403.9	405.5
	Area (%)	36.4	12.0	23.8	20.3	4.0	3.6
Pt/Fe-N-C_{Aero}	Peak (eV)	398.5	400.0	401.1	402.5	405.6	400.5
	Area (%)	36.6	15.8	23.4	17.5	6.8	0.0

Supplementary Table 6 | Relative atomic percentages of different chemical groups obtained by peak-fitting of the Pt 4*f* regions of the X-ray photoelectron spectra recorded on Pt/C TKK, Pt/N–C_{Aero} and Pt/Fe–N–C_{Aero}. Three contributions were considered to fit Pt 4*f* region orbitals 7/2 and 5/2: Pt^0, Pt^{2+} and Pt^{4+}, corresponding to 71.7 – 72.2 eV and 75.0 – 75.4 eV, 72.7 – 73.2 eV and 76.3 – 76.7 eV, 75.0 – 75.4 eV and 78.7 eV binding energies, respectively, according to the literature.[15–18]

	Pt 4*f*	**7/2**	**5/2**	**7/2**	**5/2**	**7/2**	**5/2**
**Catalysts**	**Info**	**Pt^0**	**Pt^0**	**Pt^{2+}**	**Pt^{2+}**	**Pt^{4+}**	**Pt^{4+}**
**Pt/C - TKK**	**Peak (eV)**	71.7	75.0	72.9	76.2	74.7	78.0
	**Area (%)**	33.4	25.3	13.3	10.8	8.3	8.9
**Pt/N-C_{Aero}**	**Peak (eV)**	72.1	75.4	73.3	76.6	75.1	78.4
	**Area (%)**	31.2	20.4	15.6	13.1	11.5	8.3
**Pt/Fe-N-C_{Aero}**	**Peak (eV)**	72.2	75.5	73.8	77.1	75.5	78.8
	**Area (%)**	31.5	20.6	16.0	12.9	10.9	8.1

Supplementary Table 7 | Normalized mass activity for the ORR measured at 0.8 V ($MA_{@0.8V}$) for the Fe–N–C_{Aero} and Pt/Fe–N–C_{Aero} catalysts in the different accelerated stress (AST) conditions. The $MA_{@0.8V}$ values were obtained from *iR*-drop and background-corrected ORR polarization curves at v = 2 mV s^{-1} and ω = 1600 rpm, T = 25 °C in O_2-saturated 0.1 M H_2SO_4. The catalyst loading was 400 μg_{powder} cm^{-2}_{geo}. Normalization is achieved by dividing the final $MA_{@0.8V}$ value (following a specific AST) by the initial value (first polarization curve – prior to any AST). The term "N/A" it is used here as "not applicable".

AST	Atmosphere	Temperature	Upper Potential Limit	Normalized MA at 0.8 V	
				**Fe-N-C_{Aero}**	**Pt/Fe-N-C_{Aero}**
**1**	**Ar**	**25 °C**	**0.92 V**	0.85 ± 0.06	0.73 ± 0.05
**2**			**1.00 V**	0.77 ± 0.03	0.71 ± 0.02
**3**		**80 °C**	**0.92 V**	0.53 ± 0.04	0.47 ± 0.16
**4**			**1.00 V**	0.36 ± 0.01	0.50 ± 0.01
**5**	**O_2**	**25 °C**	**0.92 V**	0.55 ± 0.03	0.70 ± 0.11
**6**			**1.00 V**	0.47 ± 0.08	0.65 ± 0.05
**7**		**80 °C**	**0.92 V**	0.22 ± 0.01	0.49 ± 0.12
**8**			**1.00 V**	0.21 ± 0.08	0.41 ± 0.15
**9**	**Air**	**25 °C**	**N/A**	0.83 ± 0.07	0.97 ± 0.02
**10**		**80 °C**	**N/A**	0.74 ± 0.01	0.69 ± 0.08

Supplementary Table 8 | Fe content measured by energy-dispersive X-ray spectroscopy at different stages of AST-7 for Fe–N–C_{Aero}. 'Fresh' represents the pristine powder; 'Acid exposure' represents the electrode (RDE tip with the catalyst ink deposited) after immersion for 17 h in 0.1 M H_2SO_4 at room temperature. For EoL, different zones were observed on the sample and LD-Fe and HD-Fe refer to low-density and high-density Fe regions, respectively.

**Catalyst**	**Atomic percentage**				
	**Metal**	**Fresh**	**Acid exposure**	**EoL**	
				**LD-Fe**	**HD-Fe**
**Fe-N-C_{Aero}**	**Fe**	0.25±0.04	0.09±0.02	0.04±0.02	1.10±0.90
	**Pt**	0.21±0.19	0.10±0.06	0.16±0.12	N/A

Supplementary Table 9 | Fe content measured by energy-dispersive X-ray spectroscopy at different stages of AST-7 for Pt/Fe–N–C_{Aero}. 'Fresh' represents the pristine powder; 'Acid exposure' represents the electrode (RDE tip with the catalyst ink deposited) after immersion for 17 h in 0.1 M H_2SO_4 at room temperature. No high-density Fe regions were observed in this case.

**Catalyst**	**Atomic percentage**			
	**Metal**	**Fresh**	**Acid exposure**	**EoL**
**Pt/Fe-N-C_{Aero}**	**Fe**	0.21±0.04	0.07±0.03	0.07±0.05
	**Pt**	0.21±0.19	0.10±0.06	0.16±0.12

Supplementary Table 10 | Parameters derived from the fittings of BoT and EoT Mössbauer spectra measured at -268 °C of the $Fe_{0.5}$ and Pt/$Fe_{0.5}$ cathodes before and after potential hold at 0.5 V (uncorrected for *iR*-drop) in PEMFC for 50 h: *RA* (%), *IS* ($mm\cdot s^{-1}$), *QS* ($mm\cdot s^{-1}$), *LW* ($mm\cdot s^{-1}$) and *H* (Tesla) of each component.

(*): stands for superparamagnetic.

	**Component**	***RA*** **%**	***IS*** **$mm\cdot s^{-1}$**	***QS*** **$mm\cdot s^{-1}$**	***LW*** **$mm\cdot s^{-1}$**	***H*** **Tesla**	**Assignment**
**$Fe_{0.5}$-cathode (BoT)**	D1	43	0.53	1.00	0.81	-	HS O-$Fe^{3+}N_4$
	D2	49	0.53	2.70	1.50	-	LS or MS $Fe^{2+}N_4$
	Sextet 1	8	0.60	-	1.10	44.0	spm(*) HS Fe_2O_3
**$Fe_{0.5}$-cathode (EoT)**	D1	6	0.51	0.82	0.50	-	HS O-$Fe^{3+}N_4$
	D2	51	0.55	2.36	1.90	-	LS or MS $Fe^{2+}N_4$
	Sextet 1	26	0.50	-	1.06	48.6	HS Fe_2O_3
	Sextet 2	17	0.43	-	1.06	56.2	spm(*) HS Fe_2O_3
**Pt/$Fe_{0.5}$-cathode (BoT)**	D1	47	0.50	0.99	0.70	-	HS O-$Fe^{3+}N_4$
	D2	53	0.52	2.55	1.47	-	LS or MS $Fe^{2+}N_4$
**Pt/$Fe_{0.5}$-cathode (EoT)**	D1	14	0.50	0.86	0.62	-	HS O-$Fe^{3+}N_4$
	D2	49	0.57	2.21	1.79	-	LS or MS $Fe^{2+}N_4$
	Sextet 1	18	0.49	-	1.03	51.6	spm(*) HS Fe_2O_3
	Sextet 2	6	0.49	-	0.76	58.2	spm(*) HS Fe_2O_3
	Sextet 3	13	0.49	-	1.03	46.2	spm(*) HS Fe_2O_3

Supplementary Table 11 | The Fe-N distances (in Å) as a function of the total spin on ferrous FeN_4C_x (x = 10 and 12) periodic models, without and with adsorbed Pt atom. The considered periodic structures are shown in Supplementary Fig. 24. The scalar (1, 2, 3, 4) after FeN indicates which exact N atom is considered for the Fe-N distance, while the moiety always has the FeN_4 stoichiometry.

Total spin	FeN1	FeN2	FeN3	FeN4	FeN1	FeN2	FeN3	FeN4
	**FeN_4C_{10}**				**Pt / FeN_4C_{10}**			
**0**	1.879	1.879	1.879	1.879	1.886	1.885	1.882	1.882
**1**	1.884	1.884	1.884	1.884	1.886	1.886	1.884	1.885
**2**	1.892	1.892	1.892	1.892	1.891	1.890	1.889	1.889
**3**	1.947	1.947	1.947	1.947	1.885	1.885	1.895	1.895
	**FeN_4C_{12}**				**Pt / FeN_4C_{12}**			
**0**	2.007	2.007	2.007	2.007	2.008	2.006	2.009	2.007
**1**	2.014	2.014	2.014	2.014	2.016	2.017	2.015	2.018
**2**	2.012	2.012	2.012	2.012	2.013	2.015	2.012	2.016
**3**	2.064	2.064	2.064	2.064	2.066	2.066	2.064	2.067

Supplementary Table 12 | The same as Supplementary Table 13 but the Fe-N distance was averaged on the four Fe-N distances.

Total spin	Average Fe-N distance / Å	Average Fe-N distance / Å
	**FeN_4C_{10}**	**Pt / FeN_4C_{10}**
**0**	1.879	1.884
**1**	1.884	1.885
**2**	1.892	1.890
**3**	1.947	1.890
	**FeN_4C_{12}**	**Pt / FeN_4C_{12}**
**0**	2.007	2.007
**1**	2.014	2.016
**2**	2.012	2.014
**3**	2.064	2.066

Supplementary Table 14 | The Fe-N and Fe-O distances (in Å) as a function of the total spin on ferric FeN_4C_x (x = 10 and 12) periodic models, without and with adsorbed Pt atom. The considered periodic structures are shown in Supplementary Fig. 24. The scalar (1, 2, 3, 4) after FeN indicates which exact N atom is considered for the Fe-N distance, while the moiety always has the FeN_4 stoichiometry.

Total Spin	FeN1	FeN2	FeN3	FeN4	FeO	FeN1	FeN2	FeN3	FeN4	FeO
	**OOH / FeN_4C_{10}**					**OOH / Pt / FeN_4C_{10}**				
**1/2**	1.901	1.905	1.903	1.900	1.782	1.902	1.901	1.897	1.899	1.786
**3/2**	1.922	1.932	1.929	1.923	1.890	1.901	1.907	1.905	1.900	1.790
**5/2**	2.047	2.046	2.046	2.047	1.880	1.923	1.919	1.919	1.923	1.911
	**OOH / FeN_4C_{12}**					**OOH/ Pt / FeN_4C_{12}**				
**1/2**	2.018	2.024	2.036	2.030	1.763	2.029	2.037	2.018	2.027	1.764
**3/2**	2.020	2.026	2.039	2.033	1.761	2.032	2.037	2.022	2.031	1.762
**5/2**	2.044	2.049	2.034	2.024	1.922	2.028	2.037	2.044	2.051	1.924

Supplementary Table 154 | The same as Supplementary Table 13 but the Fe-N distance was averaged on the four Fe-N distances.

Total Spin	Average Fe-N distance / Å	FeO	Average Fe-N distance / Å	FeO
	**OOH / FeN_4C_{10}**		**OOH / Pt / FeN_4C_{10}**	
**1/2**	1.902	1.782	1.900	1.786
**3/2**	1.926	1.890	1.903	1.790
**5/2**	2.047	1.880	1.921	1.911
	**OOH / FeN_4C_{12}**		**OOH / Pt / FeN_4C_{12}**	
**1/2**	2.027	1.763	2.028	1.764
**3/2**	2.029	1.761	2.030	1.762
**5/2**	2.038	1.922	2.040	1.924

Supplementary Table 16 | The same models and electronic states as Supplementary Table 13 but reporting the out-of-plane displacement of Fe above the N_4 plane. As the four nitrogen atoms are not always perfectly co-planar, the average of their vertical positions was used to report the Fe displacement.

Total Spin	Out-of-plane displacement of Fe above the N_4 plane / Å	Out-of-plane displacement of Fe above the N_4 plane / Å
	**OOH / FeN_4C_{10}**	**OOH / Pt / FeN_4C_{10}**
**1/2**	0.019	0.019
**3/2**	0.024	0.018
**5/2**	0.049	0.020
	**OOH / FeN_4C_{12}**	**OOH / Pt / FeN_4C_{12}**
**1/2**	0.013	0.012
**3/2**	0.014	0.013
**5/2**	0.013	0.012

Supplementary Information References

1. Jaouen, F. O2 Reduction Mechanism on Non-Noble Metal Catalysts for PEM Fuel Cells. Part II: A Porous-Electrode Model To Predict the Quantity of H2O2 Detected by Rotating Ring-Disk Electrode. *J. Phys. Chem. C* **113**, 15433–15443 (2009).
2. Li, J. *et al.* Identification of durable and non-durable FeNx sites in Fe–N–C materials for proton exchange membrane fuel cells. *Nat. Catal.* **4**, 10–19 (2021).
3. Shao, M., Peles, A. & Shoemaker, K. Electrocatalysis on Platinum Nanoparticles: Particle Size Effect on Oxygen Reduction Reaction Activity. *Nano Lett.* **11**, 3714–3719 (2011).
4. Bae, G. *et al.* Quantification of Active Site Density and Turnover Frequency: From Single-Atom Metal to Nanoparticle Electrocatalysts. *JACS Au* **1**, 586–597 (2021).
5. Jiao, L. *et al.* Chemical vapour deposition of Fe–N–C oxygen reduction catalysts with full utilization of dense Fe–N4 sites. *Nat. Mater.* **20**, 1385–1391 (2021).
6. Mehmood, A. *et al.* High loading of single atomic iron sites in Fe–NC oxygen reduction catalysts for proton exchange membrane fuel cells. *Nat. Catal.* **5**, 311–323 (2022).
7. Reichmann, I. *et al.* Scanning Gas Diffusion Electrode Setup for Real-Time Analysis of Catalyst Layers. *ACS Meas. Sci. Au* (2024) doi:10.1021/acsmeasuresciau.4c00018.
8. Cançado, L. G. *et al.* Quantifying Defects in Graphene via Raman Spectroscopy at Different Excitation Energies. *Nano Lett.* **11**, 3190–3196 (2011).
9. Härmas, R. *et al.* Carbide-Derived Carbons: WAXS and Raman Spectra for Detailed Structural Analysis. *C* **7**, 29 (2021).
10. Sadezky, A., Muckenhuber, H., Grothe, H., Niessner, R. & Pöschl, U. Raman microspectroscopy of soot and related carbonaceous materials: Spectral analysis and structural information. *Carbon* **43**, 1731–1742 (2005).
11. Lafuerza, S., Carlantuono, A., Retegan, M. & Glatzel, P. Chemical Sensitivity of Kβ and Kα X-ray Emission from a Systematic Investigation of Iron Compounds. *Inorg. Chem.* **59**, 12518–12535 (2020).

12. Artyushkova, K. Misconceptions in interpretation of nitrogen chemistry from x-ray photoelectron spectra. *J. Vac. Sci. Technol. A* **38**, 031002 (2020).
13. Artyushkova, K. *et al.* Correlations between Synthesis and Performance of Fe-Based PGM-Free Catalysts in Acidic and Alkaline Media: Evolution of Surface Chemistry and Morphology. *ACS Appl. Energy Mater.* **2**, 5406–5418 (2019).
14. Artyushkova, K. *et al.* Role of Surface Chemistry on Catalyst/Ionomer Interactions for Transition Metal–Nitrogen–Carbon Electrocatalysts. *ACS Appl. Energy Mater.* **1**, 68–77 (2018).
15. Croy, J. R., Mostafa, S., Heinrich, H. & Cuenya, B. R. Size-selected Pt Nanoparticles Synthesized via Micelle Encapsulation: Effect of Pretreatment and Oxidation State on the Activity for Methanol Decomposition and Oxidation. *Catal. Lett.* **131**, 21–32 (2009).
16. Vedrine, J. C., Dufaux, M., Naccache, C. & Imelik, B. X-ray photoelectron spectroscopy study of Pd and Pt ions in type Y-zeolite. Electron transfer between metal aggregates and the support as evidenced by X-ray photoelectron spectroscopy and electron spin resonance. *J. Chem. Soc., Faraday Trans. 1* **74**, 440 (1978).
17. Xu, J. *et al.* One-pot synthesis of three-dimensional platinum nanochain networks as stable and active electrocatalysts for oxygen reduction reactions. *J. Mater. Chem.* **22**, 13585–13590 (2012).
18. Ma, J. *et al.* Electronic interaction between platinum nanoparticles and nitrogen-doped reduced graphene oxide: effect on the oxygen reduction reaction. *J. Mater. Chem. A* **3**, 11891–11904 (2015).
