# Supplementary material for: Evidence for the stabilization of FeN4 sites by Pt particles during acidic oxygen reduction
Source: Nat Commun. 2025 Jul 11;16:6404. doi: 10.1038/s41467-025-61806-x (PMC12246215; doi:10.1038/s41467-025-61806-x)
Supplement: Supplementary file 2 — Description of Additional Supplementary Files [file 41467_2025_61806_MOESM2_ESM.pdf]

Description of Additional Supplementary Files

File name: Supplementary Data 1

Description: The atomic coordinates of the optimized FeN_4C_{10} model with $S_{tot} = 0$

File name: Supplementary Data 2

Description: the atomic coordinates of the optimized FeN_4C_{10} model with $S_{tot} = 1$

File name: Supplementary Data 3

Description: the atomic coordinates of the optimized FeN_4C_{10} model with $S_{tot} = 2$

File name: Supplementary Data 4

Description: the atomic coordinates of the optimized FeN_4C_{10} model with $S_{tot} = 3$

File name: Supplementary Data 5

Description: The atomic coordinates of the optimized OOH/FeN_4C_{10} model with $S_{tot} = 1/2$

File name: Supplementary Data 6

Description: The atomic coordinates of the optimized OOH/FeN_4C_{10} model with $S_{tot} = 3/2$

File name: Supplementary Data 7

Description: The atomic coordinates of the optimized OOH/FeN_4C_{10} model with $S_{tot} = 5/2$

File name: Supplementary Data 8

Description: The atomic coordinates of the optimized $OOH/Pt/FeN_4C_{10}$ model with $S_{tot} = 1/2$

File name: Supplementary Data 9

Description: The atomic coordinates of the optimized $OOH/Pt/FeN_4C_{10}$ model with $S_{tot} = 3/2$

File name: Supplementary Data 10

Description: The atomic coordinates of the optimized $OOH/Pt/FeN_4C_{10}$ model with S_{tot} = 5/2

File name: Supplementary Data 11

Description: The atomic coordinates of the optimized Pt/FeN_4C_{10} model with S_{tot} = 0

File name: Supplementary Data 12

Description: The atomic coordinates of the optimized Pt/FeN_4C_{10} model with S_{tot} = 1

File name: Supplementary Data 13

Description: The atomic coordinates of the optimized Pt/FeN_4C_{10} model with S_{tot} = 2

File name: Supplementary Data 14

Description: The atomic coordinates of the optimized Pt/FeN_4C_{10} model with S_{tot} = 3

File name: Supplementary Data 15

Description: The atomic coordinates of the optimized FeN_4C_{12} model with S_{tot} = 0

File name: Supplementary Data 16

Description: The atomic coordinates of the optimized FeN_4C_{12} model with S_{tot} = 1

File name: Supplementary Data 17

Description: The atomic coordinates of the optimized FeN_4C_{12} model with S_{tot} = 2

File name: Supplementary Data 18

Description: The atomic coordinates of the optimized FeN_4C_{12} model with S_{tot} = 3

File name: Supplementary Data 19

Description: The atomic coordinates of the optimized OOH/FeN_4C_{12} model with S_{tot} = 1/2

File name: Supplementary Data 20

Description: The atomic coordinates of the optimized OOH/FeN_4C_{12} model with S_{tot} = 3/2

File name: Supplementary Data 21

Description: The atomic coordinates of the optimized OOH/FeN_4C_{12} model with S_{tot} = 5/2

File name: Supplementary Data 22

Description: The atomic coordinates of the optimized OOH/Pt/FeN_4C_{12} model with S_{tot} = 1/2

File name: Supplementary Data 23

Description: The atomic coordinates of the optimized OOH/Pt/FeN_4C_{12} model with S_{tot} = 3/2

File name: Supplementary Data 24

Description: The atomic coordinates of the optimized OOH/Pt/FeN_4C_{12} model with S_{tot} = 5/2

File name: Supplementary Data 25

Description: The atomic coordinates of the optimized Pt/FeN_4C_{12} model with S_{tot} = 0

File name: Supplementary Data 26

Description: The atomic coordinates of the optimized Pt/FeN_4C_{12} model with S_{tot} = 1

File name: Supplementary Data 27

Description: The atomic coordinates of the optimized Pt/FeN_4C_{12} model with S_{tot} = 2

File name: Supplementary Data 28

Description: The atomic coordinates of the optimized Pt/FeN_4C_{12} model with S_{tot} = 3
